# Designing and mapping cascade catalysis pathway for balanced polysulfide conversion in Li-S batteries

**DOI:** 10.1038/s41467-026-74160-3

**Published:** 2026-07-01

**Authors:** Leyuan Zhang, Dongfang Cheng, Pu Zhang, David G. Hopkinson, Zhaozong Wang, Ao Zhang, Chen Li, Ran Wang, Rongli Liu, Christopher S. Allen, Johanna Nelson Weker, Yu Huang, Philippe Sautet, Xiangfeng Duan

**Affiliations:** 1 Department of Chemistry and Biochemistry, University of California, Los Angeles, Los Angeles, CA USA; 2 Department of Materials Science and Engineering, University of California, Los Angeles, Los Angeles, CA USA; 3 Department of Chemical and Biomolecular Engineering, University of California, Los Angeles, Los Angeles, CA USA; 4 SLAC, National Accelerator Laboratory, Menlo Park, CA USA; 5 Electron Physical Science Imaging Centre, Diamond Light Source, Harwell Science and Innovation Campus, Didcot, UK; 6 Department of Materials, University of Oxford, Oxford, UK; 7 California NanoSystems Institute, University of California, Los Angeles, Los Angeles, CA USA

**Keywords:** Batteries, Electrocatalysis, Batteries, Batteries

## Abstract

Lithium–sulfur batteries are fundamentally constrained by the sluggish 16-electron sulfur reduction reaction. Electrocatalytic sulfur reduction reaction is inherently complex, involving multiple lithium polysulfide intermediates (Li_2_S_n_, *n* = 2–8), each with distinct adsorption and activation requirements, leading to unbalanced polysulfide conversion and severe shuttle effect. Although cascade catalysis has been proposed as a potential solution, the precise pathway and its mechanistic role in regulating polysulfide conversion remain elusive. Here we elucidate and experimentally validate the complete cascade pathway of sulfur reduction on Fe,N,S-codoped holey graphene as a model catalyst. Density functional theory reveals that Fe sites preferentially bind and activate long-chain polysulfides, while N,S-C sites accelerate the conversion of Li_2_S_4_ to Li_2_S_2_/Li_2_S. Such site-specific synergy balances sulfur reduction kinetics and suppresses polysulfide accumulation. Combined kinetic analysis and operando Raman spectroscopy directly reveal how synergistic cascade catalysis governs the reaction pathway, modulates key intermediates, and enables balanced polysulfide conversion. Together, these results establish cascade catalysis as a mechanism-driven design strategy for lithium–sulfur battery electrodes, where regulation of the reaction pathway suppresses polysulfide shuttling and enables enhanced cycling stability.

## Abstract (web summary)

Li-S batteries suffer from unbalanced polysulfide conversion and severe polysulfide shuttling. Here, the authors reveal that chemically distinct active sites in multi-heteroatom-doped holey graphene cooperatively orchestrate a cascade pathway of sulfur reduction reaction, enabling balanced polysulfide conversion.

## Introduction

Sulfur represents a promising positive electrode material for next-generation lithium batteries for its high theoretical capacity (1675 mAh g^−1^), natural abundance, and low cost^1–4^, making it an attractive option for large-scale energy storage applications. However, the complex 16-electron sulfur reduction reaction (SRR) poses persistent challenges for practical lithium-sulfur (Li–S) batteries (Supplementary Table 1)^5–7^. In general, SRR involves a multistep conversion process from solid S_8_ to Li_2_S_2_/Li_2_S solids, during which a series of soluble lithium polysulfides (LiPSs, e.g., Li_2_S_8_, Li_2_S_6_, Li_2_S_4_) can form and shuttle across the separator to the Li metal negative electrode, resulting in the loss of active sulfur and Li metal^8–10^. It is recognized that the conversion from soluble LiPSs to insoluble Li_2_S_2_/L_2_S is kinetically sluggish, leading to LiPS accumulation and shuttling that could seriously undermine the cycling stability^11^.

Considerable efforts have been made on accelerating SRR kinetics and mitigating the LiPS shuttle effect, for example, by designing specific host materials and catalysts to physically and/or chemically confine LiPSs and electrocatalytically accelerate their conversion^11–38^. In particular, heteroatom-doped holey graphene frameworks (HGFs) with highly interconnected holey graphene sheets and heteroatom-doping sites^39,40^ offer an attractive platform for sulfur electrodes (Supplementary Fig. 1). This highly interconnected graphene network with electrocatalytically active sites can simultaneously facilitate rapid ion and electron transport, and enhance the sulfur reaction kinetics, thus mitigating the LiPS shuttle effect and improving sulfur utilization even at high sulfur loadings^39,41,42^. Additionally, the high-density dopants on the large surface area of HGFs promote uniform Li_2_S deposition, which is crucial for preventing the solid-state product from passivating catalytic sites in the battery catalysis.

Current catalyst designs, which typically feature a single type of active site, face inherent limitations in catalyzing the multistep SRR involving diverse LiPS intermediates (Li_2_S_n_, *n* = 2–8). As these intermediates exhibit markedly different binding affinities and activation requirements, a single active site cannot efficiently facilitate all reaction steps, resulting in unbalanced LiPS conversion. This imbalance leads to polysulfide accumulation and triggers parasitic side reactions, such as deleterious comproportionation reactions that produce electrochemically passive or less active LiPS species (e.g., Li_2_S_6_)^43^, further exacerbating polysulfide shuttling and compromising cycling stability. SRR catalysts incorporating multiple active centers have been reported, and the concept of cascade catalysis has been invoked to interpret the observed differences in battery performance^44,45^. However, current studies of SRR cascade catalysis remain largely limited to phenomenological correlations with overall battery performance and DFT-derived adsorption energies. The studies to date did not establish how distinct active sites cooperatively mediate sequential reaction steps, map the complete reaction pathway, or experimentally verify how cascade reactions govern the conversion of specific intermediates. Addressing these gaps is essential to establish cascade catalysis as a rational design strategy for electrode materials in Li–S batteries.

Herein, we employ multi-heteroatom-doped HGFs as model cascade catalysts to elucidate the fundamental role of cascade reaction pathways in facilitating balanced SRR. By incorporating metal (Fe) and non-metal dopants (N, S) in HGFs, we introduce two distinct yet synergistic active sites: Fe sites that bind long-chain LiPSs and facilitate their conversion, and N, S–C sites that accelerate subsequent conversion of short-chain LiPSs. Computational analyses map out the full reaction network and delineate the cascade reaction pathway, which is directly validated by comprehensive kinetic measurements and operando Raman spectroscopy. Together, these findings establish a mechanistic foundation for cascade catalysis in SRR, demonstrating how the interplay between distinct active sites drives balanced LiPS conversion and suppresses polysulfide accumulation, which effectively mitigates the shuttle effect and enables markedly improved cycling stability.

## Results

### The design of HGFs with synergistic dual active sites

An optimal catalyst should strike a balance in its adsorption affinities, exhibiting strong enough adsorption to stabilize the reactant and facilitate its activation, while maintaining a sufficiently weak affinity for the product to ensure the efficient desorption, thereby freeing the active sites for subsequent catalytic cycles. Such a balance is critical for maintaining high catalytic turnover and minimizing product inhibition, which can otherwise reduce overall catalytic efficiency. This general catalyst design principle presents a fundamental dilemma for the multistep, 16-electron SRR, where multiple reaction intermediates exhibit distinct binding affinities at a given catalytic site. Balancing the adsorption energies for all intermediates becomes increasingly challenging, as strong binding may stabilize certain intermediates but hinder the desorption of others. This could lead to kinetic bottlenecks and limit overall catalytic efficiency.

**Fig. 1:**
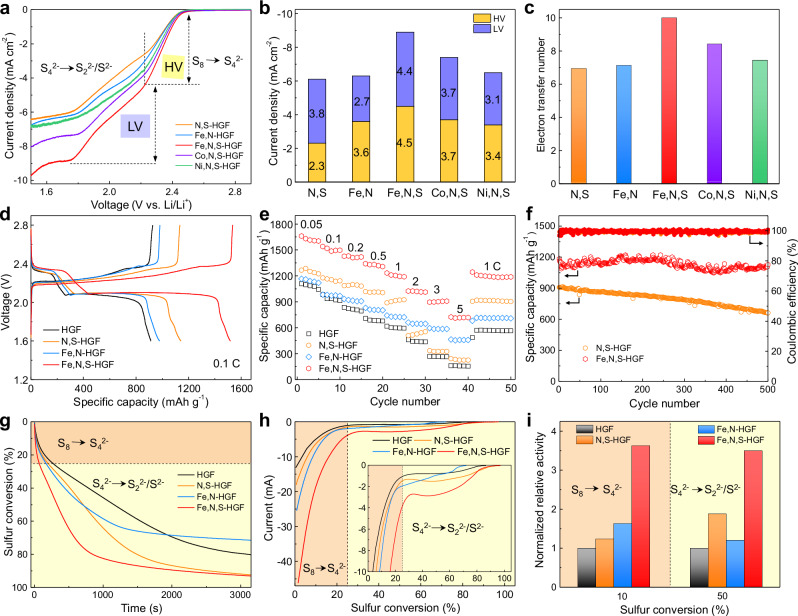
Synergetic strategy of heteroatom-doped HGF host for SRR. **a**, **b** Schematic illustration of single-site (**a**) and dual-site (**b**) electrocatalytic SRR process, highlighting the synergistic cascade catalysis on dual active sites. This cascade process restrains long-chain LiPSs on the catalyst surface and facilitates the balanced conversion of LiPSs into the final product, thus suppressing the comproportionation reaction and mitigating the LiPS shuttle effect.

For example, the 16-electron SRR typically consists of multiple cascade reactions: S_8_ → Li_2_S_8_ → Li_2_S_4_ → Li_2_S_2_ → Li_2_S, converting long-chain LiPSs (e.g., Li_2_S_8_) to intermediate or short-chain LiPSs (Li_2_S_4_, Li_2_S_2_) and eventually to Li_2_S. The efficient desorption of intermediate products (e.g., Li_2_S_4_ in the Li_2_S_8_ → Li_2_S_4_ conversion) represents an essential prerequisite for optimal catalytic performance. However, recent studies suggest that desorbed Li_2_S_4_ can undergo a comproportionation reaction with Li_2_S_8_ in the electrolyte to form highly soluble Li_2_S_6_ (Li_2_S_8_ + Li_2_S_4_ → 2Li_2_S_6_) (Fig. 1a). This Li_2_S_6_ has fundamentally sluggish down-conversion kinetics, which kinetically traps the reaction and contributes significantly to the undesirable accumulation and shuttle effect of LiPS (see Fig. 1a)^43^.

Designing catalysts that effectively restrain reaction intermediates on the solid catalyst surface could bypass the comproportionation reaction, mitigating LiPS accumulation and the shuttle effect. However, such retention may passivate the catalytic sites over time and prevent continued reduction and down conversion^46^. Therefore, an ideal SRR catalyst design should not only accelerate the conversion of long-chain LiPS (e.g., Li_2_S_8_ → Li_2_S_4_), but also efficiently restrain the intermediates, such as Li_2_S_4_ on the solid catalyst surface and facilitate its further down-conversion (e.g., Li_2_S_4_ → Li_2_S_2_/Li_2_S), without desorption or diffusion into the electrolyte. To this end, by simultaneously incorporating non-metal (e.g., N, S) and metal (e.g., Fe, Co, Ni) based heteroatom dopants into HGF, we introduce two distinct binding sites that work synergistically: one adsorbing long-chain LiPS and facilitating their conversion, while the other facilitating subsequent conversion of short-chain LiPS (Fig. 1b). This mechanism balances the LiPS conversion process, suppresses the dissolution of Li_2_S_4_ and prevents the formation of highly soluble Li_2_S_6_, thus effectively mitigating the associated shuttle effect.

### HGFs with synergistic active sites

A series of heteroatom-doped HGFs were designed as the model catalysts to explore the synergistic electrocatalytic SRR, including the non-doped HGF, N, S-HGF, Fe, N-HGF, Fe, N, S-HGF, Co, N, S-HGF, and Ni, N, S-HGF. The hierarchical HGF architecture is formed by the hydrothermal reaction, showing a three-dimensional (3D) graphene framework structure for fast electron transport/transfer, along with an interconnected pore structure for efficient Li^+^ diffusion (Supplementary Fig. 2). Abundant edge sites are available for heteroatom incorporation in the holey graphene structure. Transmission electron microscopy (TEM) and associated energy dispersive spectroscopy (EDS) elemental mapping revealed that heteroatom dopants are uniformly distributed within the HGF (Supplementary Fig. 3), with comparable concentrations of N or S dopants among different samples (Supplementary Fig. 4). Based on the screening result of metal-doping effect on the SRR current (see next section), we mainly conducted the structure characterization for Fe-doped samples.

**Fig. 2:**
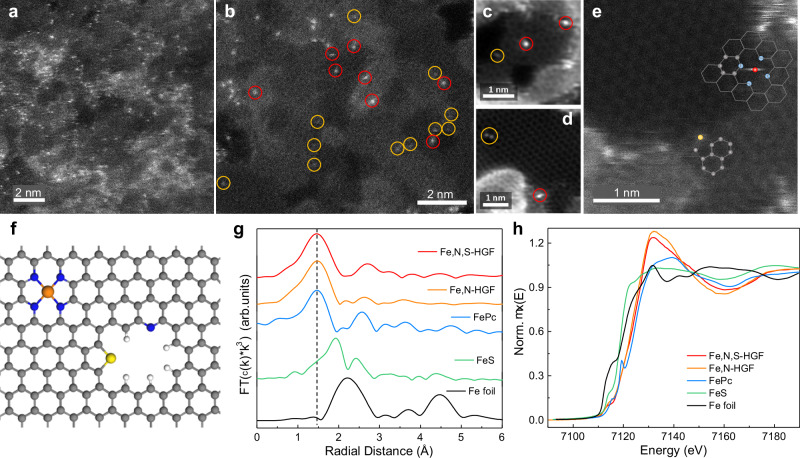
Structure characterizations of heteroatom-doped HGF for SRR. **a**, **b** ADF-STEM images of Fe, N, S-HGF (**a**) and an enlarged image (**b**) showing the isolated iron (red circle)/sulfur (yellow circle) dopants in the graphene matrix. **c**, **d** ADF-STEM images of monolayer regions showing sulfur dopant at edge (yellow circle), and single iron dopants (red circle) (**c**), and sulfur dopant dimer at a nanopore (yellow circle) and iron dopant at bilayer edge (red circle) (**d**). N is indistinguishable from the carbon matrix. **e** A high-resolution TEM image shows the visualization of the atomic dopants of Fe and S embedded in the graphene lattice. The overlaid schematics represent the models of atomic structure. **f** The schematic representation of the possible atomic structure in Fe, N, S-HGF. C atoms are black; N atoms are blue; Fe atom is orange; S atom is yellow. **g**, **h** Fe K-edge FT-EXAFS (**g**) and XANES (**h**) of Fe, N, S-HGF and Fe, N-HGF with the reference Fe foil, FeS and FePc (iron phthalocyanine).

We have conducted detailed structural analysis, including the annular dark-field scanning TEM (ADF-STEM), Fe K-edge extended X-ray absorption fine structure (EXAFS), and X-ray absorption near-edge structure (XANES), to probe the bonding structure and valence state of Fe-doped HGF samples (Fe, N, S-HGF and Fe, N-HGF). The ADF-STEM study provides a positive contrast, where the measured pixel intensity is approximately proportional to the square of the atomic number. It reveals that single atom doping dominates in both Fe, N- and Fe, N, S-HGFs (Fig. 2a, Supplementary Figs. 5, 6). For the Fe, N, S-HGF, both Fe and S doping are visible and apparently do not tend to co-locate next to each other (Fig. 2b–e). In Fig. 2c, d, the carbon lattice provides low contrast but is identifiable through its characteristic honeycomb structure. The brightest points within the lattice are consistent with a heavier species (i.e., Fe), whereas points with an intensity intermediate to Fe and C are consistent with S dopants. According to the atomic structure resolved within single-layer graphene (Fig. 2e), the possible coordination configuration of the atomic structure is simulated and presented in Fig. 2f. These structural characteristics identified by STEM studies are also supported by the Fe K-edge EXAFS, which shows the first large peak at around 1.48 Å for both Fe, N, S-HGF and Fe, N-HGF, corresponding to the predominant interaction between Fe and lighter elements (C, N, or O) (Fig. 2g). The highly comparable EXAFS data observed in the Fe, N, S-HGF and Fe, N-HGF samples (Supplementary Fig. 7) suggests a highly similar local Fe coordination environment for both samples, which is also similar to that of Fe, N-HGF reported previously^40^. In particular, the first nearest neighbor peak height remains relatively unchanged between Fe, N- and Fe, N, S-HGFs, supporting that there are no direct Fe-S interactions. This finding aligns with STEM studies that Fe and S atoms are isolated in the graphene matrix. The XANES spectra suggest that the Fe valence state is similar in Fe, N- and Fe, N, S-HGFs (Fig. 2h), showing a value between +2 and +3, as the absorption edge shifts to the right of the Fe reference foil^47^.

### The heteroatom-doping effect on electrocatalytic SRR

**Fig. 3:**
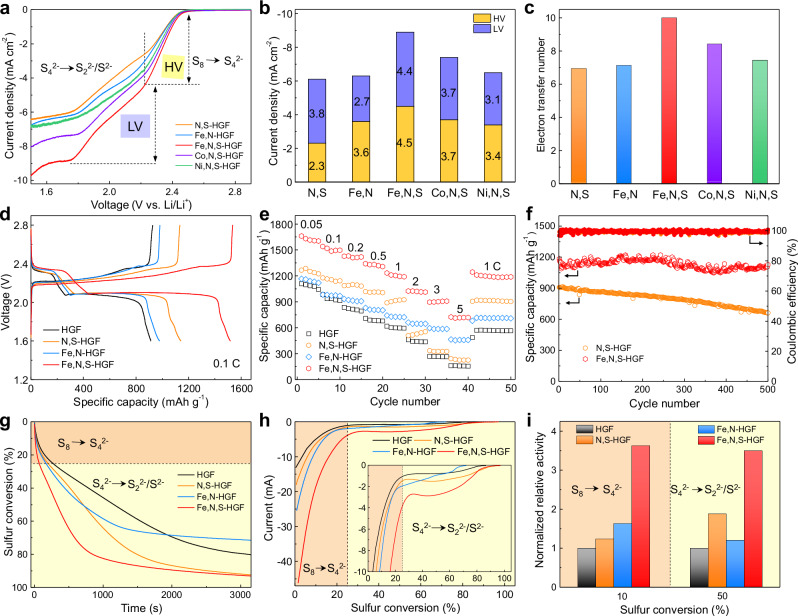
Evaluation of catalytic activity of heteroatom-doped HGFs. **a**, **b** LSV curves of heteroatom-doped HGFs for sulfur reduction (**a**), and the corresponding current densities at high-voltage (HV) and low-voltage (LV) regions, respectively (**b**). **c**, Electron transfer number comparison of heteroatom-doped HGFs during sulfur conversion. **d** Galvanostatic charge-discharge curves of heteroatom-doped HGF/sulfur electrodes at 0.1 C for Li-S cells. The polarization voltage gap represents the potential difference at the midpoint of the theoretical capacity. **e** Rate capability of heteroatom-doped HGF/sulfur electrodes from 0.05 C to 5 C. **f** Cycling stability of heteroatom-doped HGF/sulfur electrodes at a current density of 1 C. **g** Representative conversion vs. time curves (derived from chronoamperometry) of heteroatom-doped HGFs when applying the voltage of 1.8 V. **h** Current vs. conversion profiles (derived from chronoamperometry) of heteroatom-doped HGFs when applying the voltage of 1.8 V. The inset is an enlarged view of chronoamperometry discharge profiles showing the current difference in the second region. **i** Relative activity of heteroatom-doped HGFs represented by the corresponding discharge current in the chronoamperometry profiles at the specific sulfur conversion of 10% and 50%, normalized by that of HGF.

The linear sweep voltammetry (LSV) measurement on the rotating disk electrode (RDE) was carried out to directly probe the fundamental electrocatalytic SRR behavior of the heteroatom-doped HGFs in a three-electrode setup using Li metal as both the counter and reference electrode^48^ (Fig. 3a). All SRR LSV curves exhibit two specific regions separated by a transition point at around 2.2 V, in line with the reduction peak separation in CV curves, which were attributed to the S_8_ → Li_2_S_4_ and Li_2_S_4_ → Li_2_S conversions, respectively. A close examination reveals that all catalysts with N, S-C sites exhibit transition points at higher potentials than those without N, S-C sites (Fe, N-HGF) (Supplementary Fig. 8), suggesting higher onset potentials and more favorable kinetics for the Li_2_S_4_ → Li_2_S conversion when there are N, S-C sites, demonstrating the critical role of N, S-C sites for this step. The LSVs showed notably different diffusion-limited currents for different heteroatom-doped HGFs (Fe, N, S-HGF > Co, N, S-HGF > Ni, N, S-HGF > Fe, N-HGF > N, S-HGF), suggesting the introduction of metal dopants can further modify the reaction kinetics. The N, S-HGF showed a current density of ~6.1 mA cm^−2^, which is largely consistent with our previous result, while the Fe, N, S-HGF showed a greatly higher current density of ~8.9 mA cm^−2^. Intriguingly, according to the transition point (2.2 V) and end point (1.7 V), the high-voltage and low-voltage regions displayed different trends of the diffusion-limited current density: with the current following the trend of Fe, N, S (4.5) > Co, N, S (3.7) > Fe, N (3.6) > Ni, N, S (3.4) > N, S (2.3) in the high-voltage region, and the trend of Fe, N, S (4.4) > N, S (3.8) > Co, N, S (3.7) > Ni, N, S (3.1) > Fe, N (2.7) in the low-voltage region (Fig. 3b).

It is interesting to note that N, S-HGF without metal dopants showed substantially lower current destiny (2.3 mA cm^−2^) than those with Fe or Co dopants (>3.6 mA cm^−2^) in the high voltage region (S_8_ → Li_2_S_4_), suggesting the critical role of metal dopants (Fe, Co) in catalyzing long-chain LiPS conversion and the ineffectiveness of N, S-HGF in facilitating this reaction. Additionally, focusing on additional current increase in the low-voltage region (Li_2_S_4_ → Li_2_S_2_/Li_2_S), it is apparent that Fe, N-HGF without S dopants shows notably lower current density (2.7 mA cm^−2^) than those with S dopants (>3.1 mA cm^−2^), indicating the critical role of S dopants in facilitating short-chain LiPS conversion. Furthermore, the Fe, N, S-HGF showed a notably higher current (8.9 mA cm^−2^) than Fe,N-HGF (6.3 mA cm^−2^) or N, S-HGF (6.1 mA cm^−2^), underscoring the synergistic effect of the metal and non-metal doping sites on boosting the overall SRR kinetics.

In addition, the electron transfer number was calculated based on the Koutecky-Levich equation^49^, as summarized in Fig. 3c. The Fe, N, S-HGF sample shows an apparent electron transfer number of ~10.0, which is larger than those for N, S-HGF (6.9), Fe, N-HGF (7.1), Co, N, S-HGF (8.4), and Ni, N, S-HGF (7.4), suggesting Fe, N, S-HGF can promote more complete sulfur conversion into the insoluble Li_2_S_2_/Li_2_S. Overall, these LSV studies demonstrate that introducing atomic metal doping into N, S-HGF samples further enhances the electrocatalytic effect, by particularly accelerating the conversion of long-chain polysulfide (S_8_ → Li_2_S_4_).

To further study the electrochemical behavior of heteroatom-doped HGF electrodes in Li-S batteries, we assembled Li-S coin cells with the HGF/sulfur composite positive electrode and Li-metal negative electrode. All the HGF/sulfur composite electrodes exhibit two characteristic discharge plateaus at ~2.3 V and ~2.1 V, respectively (Fig. 3d). The Fe, N, S-HGF based electrode delivered a specific capacity of 1517 mAh g^−1^ at 0.1 C, with a sulfur utilization of ~91%, which is much higher than that of N, S-HGF (1142 mAh g^−1^) or Fe, N-HGF (980 mAh g^−1^), confirming the synergistic electrocatalytic effect of Fe and S dopants. The Fe, N, S-HGF also showed the smallest polarization voltage gap (158 mV), which is considerably lower than those of HGF (403 mV), N,S-HGF (237 mV), and Fe, N-HGF (308 mV), further confirming the improved electrocatalytic activity.

The Fe, N, S-HGF electrode also showed a notably higher rate capability, achieving a capacity of 907 mAh g^−1^ at 5 C ( ~ 60% capacity retention over the capacity at 0.1 C), in comparison with 19% capacity retention for N, S-HGF and with 45% capacity retention for Fe, N-HGF (Fig. 3e). In addition, the cycling test showed that the Fe, N, S-HGF achieves a capacity retention of ~95% over 500 cycles at 1 C, considerably higher than 73% for the N, S-HGF electrode (Fig. 3f, Supplementary Fig. 9). The reduced capacity fading suggests the suppressed LiPS shuttle effect in the Fe, N, S-HGF. The post-cycling characterizations confirm the structural robustness of HGF electrodes with electrochemical stability of heteroatom dopants during the electrochemical cycling (Supplementary Figs. 10–14).

To understand the influence of heteroatom dopants on the reaction kinetics of electrocatalytic SRR, we further conducted systematic chronoamperometry (i.e., potentiostatic) measurements. The chronoamperometry studies were conducted by holding the cell voltage at 1.8 V to drive sulfur reduction (discharge) or 2.8 V for Li_2_S oxidation (charge) process, and recording the current response until reaching the cut-off current (0.05 mA). As the holding voltage is sufficiently low for complete reduction or sufficiently high for complete oxidation, the responding current profile gives a sensitive measure of the overall reaction kinetics, which is mainly affected by the catalyst activity^50^.

The current-time profile reveals that the charge reaction has faster kinetics than the discharge reaction, even for the pristine HGF sample without any doping. Most of charging capacity (>90%) is completed in 400 s, corresponding to a charge rate of 9 C (Supplementary Fig. 15). The discharge profiles clearly reveal the fastest reaction kinetics in the Fe, N, S-HGF, showing the larger slope in the capacity-time curve, and reaching 50% of discharge capacity within 330 s, in comparison with 700 s for Fe, N-HGF, 710 s for N, S-HGF and 1158 s for HGF (Fig. 3g). The capacity-time plot at 1.8 V (Fig. 3g) reveals a notably different discharge rate with a turning point at approximately 25% of theoretical capacity, separating the discharge curve into two regions: the first region corresponding to the S_8_ → Li_2_S_4_, and the second region corresponding to Li_2_S_4_ → Li_2_S.

The current-capacity plot (Fig. 3h) further reveals that both the HGFs with metal dopants (Fe, N- and Fe, N, S-HGFs) exhibit significantly higher current within the first 1/4 of theoretical capacity than those without Fe dopants, highlighting the critical role of Fe dopants in accelerating the conversion of long-chain LiPSs (S_8_ → Li_2_S_4_). Beyond 1/4 capacity, Fe, N-HGF shows lower current compared to N, S-HGF, indicating the essential role of N, S doping in promoting the conversion of short-chain LiPSs (Li_2_S_4_ → Li_2_S). In particular, Fe, N-HGF has a higher kinetic current than N, S-HGF at the sulfur conversion of 10%, while N, S-HGF shows a higher kinetic current than Fe, N-HGF when the sulfur conversion is 50% (Fig. 3i), indicating the distinct role of these two different catalytic sites. In comparison, Fe, N, S-HGF show the highly improved current across the entire discharge capacity until reaching full capacity, demonstrating the synergistic effect of incorporating both metal (Fe) and non-metal (N, S) dopants on enhancing the multi-step SRR process. This approach may help suppress the desorption of Li_2_S_4_ to prevent the subsequent formation of soluble Li_2_S_6_ (major shuttling species) (Supplementary Fig. 16, Table 2).

### Theoretical investigation of the promotion effect of Fe sites

**Fig. 4:**
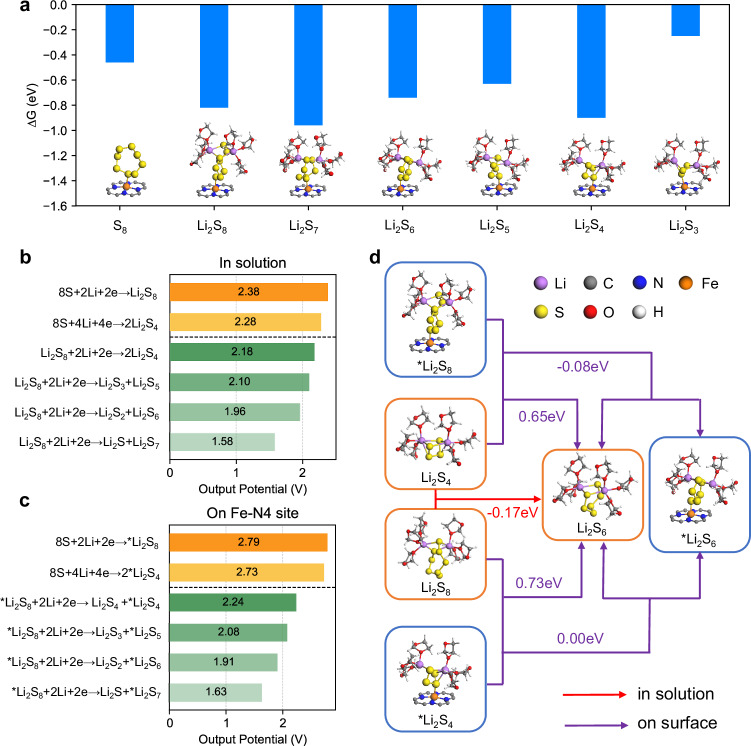
Enhanced activity of Fe sites on long-chain polysulfide conversion. **a** Adsorption free energy (∆G) of LiPSs on Fe sites. In contrast, none of these polysulfides exhibits adsorption on the N, S-HGF surface. **b**,** c** The calculated output potentials of the reaction involved in S_8_ to S_4_ conversion in solution (**b**) and on Fe sites (**c**). The reference species is always S_8_. The orange bars represent the initial transformation of S_8_ to either Li_2_S_8_ or Li_2_S_4_, while green bars correspond to the subsequent reduction of Li_2_S_8_. **d** Reaction free energies for different combinations of Li_2_S_4_ and Li_2_S_8_ (in molecular or adsorbed forms) leading to the formation of Li_2_S_6_ or adsorbed *Li_2_S_6_. Asterisks indicate surface adsorbate species. Orange and blue flames represent the molecules and adsorbed intermediates, respectively. Red and purple arrows represent the reaction occurring in solution and on the surface, respectively. The free energy analysis indicates that strong adsorption of *Li_2_S_8_ and *Li_2_S_4_ species on Fe sites can effectively suppress the comproportionation reaction of forming highly soluble Li_2_S_6_ in solution.

In our previous studies using the N, S-HGF, the conversion of S_8_ to Li_2_S_4_ predominantly occurs in the liquid phase during the discharge process^43^. Introducing Fe dopants into the N, S-HGF (i.e., Fe, N, S-HGF) significantly enhances the current density, particularly in the high-potential region associated with the S_8_ to Li_2_S_4_ conversion (Fig. 3a). This suggests that Fe sites play a critical role in the long-chain PS conversion. It is generally recognized that LiPSs can easily shuttle when dissolved in solution. We find that Fe active sites can effectively anchor LiPSs on the catalyst surface (Fig. 4a), which is beneficial for suppressing the PS shuttling. The adsorbed LiPSs largely retain a ring-like structure, similar to their configuration in solution, which is thermodynamically more stable than the chain-like structure. Upon adsorption, one sulfur atom in the Li_2_S_x_ ring interacts with the Fe site, slightly elongating the adjacent S–S bond without necessarily breaking the polysulfide ring. This interaction, illustrated in Supplementary Fig. 17, stabilizes the polysulfide structure on the Fe site. From Li_2_S_8_ to Li_2_S_4_, the adsorption strength remains relatively high due to the steric compatibility of the larger polysulfide rings and solvent shell with Fe active sites. In contrast, shorter chains such as Li_2_S_3_ exhibit weaker adsorption due to steric hindrance from the solvation shell of Li^+^, which restricts the access of S_3_ moities to the surface.

To elucidate the significant enhancement of the S_8_ to Li_2_S_4_ conversion on Fe,N,S-HGF, we compared the fundamental energetics of different polysulfide intermediates and detailed reaction pathways on Fe sites versus in solution. Beginning with the initial S_8_ reduction, computational results indicate that in solution, S_8_ is first reduced to Li_2_S_8_, yielding an output potential of 2.38 V. A direct reduction involving the transfer of four coupled Li^+^ ions and electrons to form Li_2_S_4_ results in a slightly lower output potential of 2.28 V (Fig. 4b). These findings indicate Li_2_S_8_ as a key intermediate during the early stage of SRR. As previously discussed, Fe sites can effectively capture long-chain polysulfides, potentially altering the energetics of the initial S_8_ activation. As seen in Fig. 4a, Li_2_S_8_ shows an exergonic adsorption on Fe sites at room temperature, while adsorption on N, S-C sites is very weak. As a result of the strong adsorption, the output potentials for the formation of surface-bound *Li_2_S_8_ and *Li_2_S_4_ on Fe sites are 2.79 V and 2.73 V, respectively, significantly higher than those in solution (Fig. 4c). The quantity of surface intermediates is however, too low to be clearly seen in the experimental current of Fig. 3a.

Once Li_2_S_8_ forms in solution, it undergoes a two-electron reduction to yield two Li_2_S_4_ polysulfides, as this pathway exhibits the highest output potential among various reduction pathways (Fig. 4b). On the Fe site, surface-bound *Li_2_S_8_ is most efficiently reduced to one *Li_2_S_4_, which occupies the Fe site, and simultaneously forms another Li_2_S_4_ polysulfide in solution. This process demonstrates an output potential of 2.24 V, slightly higher than the corresponding reaction in solution (2.18 V) (Fig. 4c). While the formation of two *Li_2_S_4_ on Fe sites exhibits even higher output potentials (Supplementary Fig. 18), the limited availability of Fe sites and the occupation by *Li_2_S_8_ at earlier stages suggest that this is a minor pathway. We also explored alternative intermediates such as *Li_2_S_7_, *Li_2_S_6_, and *Li_2_S_5_; however, these were found to be thermodynamically less favorable than Li_2_S_4_ (Fig. 4c, Supplementary Fig. 18). These findings indicate that the Fe site plays a pivotal role in facilitating S_8_ activation to *Li_2_S_8_ and its subsequent reduction. Furthermore, the Fe site ensures that the reduction process remains surface-confined, ultimately leading to the formation of *Li_2_S_4_ on the Fe site and one Li_2_S_4_ polysulfide in solution.

Li_2_S_6_, generated through a comproportionation reaction between Li_2_S_8_ and Li_2_S_4_ in solution, is recently recognized as a primary contributor to polysulfide shuttling^43^. This reaction is thermodynamically favorable with a reaction free energy of −0.17 eV. To investigate whether Fe sites can mitigate this reaction and thereby suppress polysulfide shuttling, we analyzed various possible combinations of reactants and products in the comproportionation process (Fig. 4d). We considered both free polysulfide states and adsorbed states of Li_2_S_8_ and Li_2_S_4_ as reactants, leading to the formation of either free Li_2_S_6_ polysulfides or adsorbed *Li_2_S_6_. Our calculations reveal that forming free Li_2_S_6_ polysulfides from either *Li_2_S_8_ + Li_2_S_4_ or Li_2_S_8_ + *Li_2_S_4_ is highly unfavorable, with reaction free energies of 0.65 eV and 0.73 eV, respectively. The formation of one Li_2_S_6_ molecule alongside one adsorbed *Li_2_S_6_ from *Li_2_S_8_ + Li_2_S_4_ and Li_2_S_8_ + *Li_2_S_4_ exhibits reaction free energies of −0.08 eV and 0.00 eV, respectively, suggesting a minor likelihood of Li_2_S_6_ polysulfide formation. However, several factors suggest that Fe sites effectively suppress this reaction. First, adsorbed *Li_2_S_6_ competes with *Li_2_S_8_ and *Li_2_S_4_ for Fe sites, as it exhibits slightly less favorable adsorption energy. This competition makes the comproportionation reaction less favorable compared to the direct reduction of S_8_ to Li_2_S_8_ and Li_2_S_8_ to Li_2_S_4_. Second, the closed-shell structures of adsorbed *Li_2_S_8_ and *Li_2_S_4_ exhibit steric hindrance, limiting their interaction with polysulfides in solution and further reducing the likelihood of comproportionation. These findings suggest that Fe sites play a critical role in mitigating the comproportionation reaction responsible for Li_2_S_6_ formation, thereby alleviating polysulfide dissolution and shuttling to a significant extent.

### Synergistic active sites enable balanced SRR

**Fig. 5:**
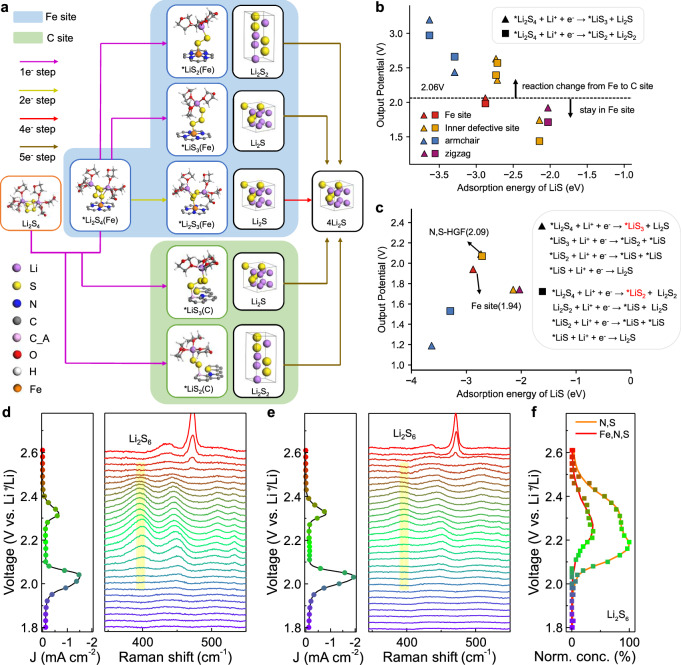
Active site change from Fe sites to C sites during SRR. **a** Possible reaction pathways for the six-electron reduction of *Li_2_S_4_. *Li_2_S_4_ can be reduced to *LiS_3_ or *LiS_2_ radicals, or to *Li_2_S_3_ molecules, with Li_2_S as the final product. Blue and green shades indicate reactions occurring on Fe sites and C sites, respectively. **b** Calculated output potentials for the initial reduction step of Li_2_S_4_ on Fe site and active C sites, plotted as a function of adsorption energy of *LiS radical. **c** Multistep output potentials for the reduction of Li_2_S_4_ to Li_2_S along the two pathways with the highest output potentials, evaluated across different active sites. Asterisks indicate surface adsorbate species. **d**, **e** CV profile and the corresponding experimental operando Raman spectra of the Raman cell with the N, S-HGF (**d**) or Fe, N, S-HGF (**e**) electrode operated at a scan rate of 0.05 mV s^−1^. The characteristic peak at around 399 cm^−1^ marked in the yellow color shade is used to quantify the Li_2_S_6_ intermediate. **f** Comparison between voltage-dependent experimental concentrations of Li_2_S_6_ derived from operando Raman spectra. The normalized concentration is calculated by dividing the concentration by the highest concentration observed in the discharge process of N, S-HGF electrodes.

As discussed before, with the reduction of *Li_2_S_8_, *Li_2_S_4_ forms and adsorbs onto the Fe site, while a portion of Li_2_S_4_ remains in the polysulfide state in solution. We thus investigated the subsequent reduction steps starting from a *Li_2_S_4_ adsorbate and Li_2_S_4_ in solution. For *Li_2_S_4_, it can either undergo reduction to LiS_2_/LiS_3_ radicals or proceed through a two-electron transfer to form *Li_2_S_3_, which remains adsorbed on the Fe site. These pathways, marked in blue (Fig. 5a), represent reduction processes that occur exclusively on the Fe site. Our calculations reveal that forming *LiS_3_ is slightly more favorable than forming *LiS_2_ on the Fe site, with output potentials of 2.06 V and 1.98 V, respectively (Fig. 5b). Conversely, the formation of *Li_2_S_3_ via a two-electron transfer step is thermodynamically unfavorable, exhibiting an output potential of 1.82 V (Supplementary Fig. 19). This is attributed to the relatively weak adsorption of short-chain polysulfide Li_2_S_3_ on the Fe site, as indicated in Fig. 4a. Based on these findings, we exclude the *Li_2_S_3_ pathway from consideration.

Another possible pathway involves *Li_2_S_4_ undergoing reduction to form *LiS_3_/*LiS_2_ radicals on adjacent N, S-C sites, which have been demonstrated in our previous studies to be highly active for Li_2_S_4_ reduction. However, if *LiS_3_ or *LiS_2_ forms on the N, S-C site and leaves the Fe site unoccupied, the reactions are highly unfavorable due to the significant energy cost of desorbing the *Li_2_S_4_ intermediate from the Fe site (Supplementary Table 3). Interestingly, Li_2_S_4_ or initially generated Li_2_S_8_ can rapidly reoccupy the Fe site and partially offset the energy cost associated with reducing *Li_2_S_4_ from the Fe site to *LiS_3_/*LiS_2_ on the N, S-C site. The output potentials for these steps on various N, S-C sites (Supplementary Fig. 20) are shown in Fig. 5b. Our results reveal that armchair-structured carbon sites exhibit relatively strong adsorption of LiS_x_ species, leading to the highest output potentials for the formation of *LiS_3_/*LiS_2_ from *Li_2_S_4_ on the Fe site due to a strong thermodynamic driving force. In contrast, the weak interaction between LiS_x_ radicals and zigzag structures results in much lower output potentials, suggesting that *LiS_3_/*LiS_2_ formation remains more favorable on Fe sites rather than diffusing to C sites. Inner defective carbon sites, which exhibit relatively strong binding with LiS_3_/LiS_2_, also indicate a thermodynamic driving force for the more reduced intermediates to be stabilized on neighboring N, S-C sites, rather than remaining bound to Fe sites. Thus, we conclude that if the adsorption free energy of *LiS_3_/*LiS_2_ radicals on N, S-C sites satisfies the condition ∆*G*(LiS_3_/LiS_2_@C) < ∆*G*(LiS_3_/LiS_2_@Fe) - ∆*G*(Li_2_S_4_@Fe), a thermodynamic driving force would favor the formation of *LiS_3_/*LiS_2_ on N, S-C sites. Such a site-to-site transfer in reactant and product stabilization would efficiently release Fe sites for sustained catalytic cycles.

To further compare the functionality of Fe sites and N,S-C sites in the Li_2_S_4_ to Li_2_S conversion process, we constructed a volcano plot to analyze this complex 6-electron transfer reaction. Building on insights from our previous work^43^ and the broad consensus that the Li_2_S_4_ to Li_2_S is rate-limiting in SRR, we considered two distinct pathways involving the formation of *LiS_3_/*LiS_2_ intermediates from *Li_2_S_4_ (Fig. 5c). Interestingly, inner defective carbon sites are positioned at the apex of the volcano plot, exhibiting the highest output potential of 2.09 V, consistent with a previous study^43^. In contrast, armchair-structured N, S-C sites, with their strong binding to radicals, and zigzag-structured N, S-C sites, with their weak binding, result in much lower output potentials. Fe sites, which demonstrate stronger radical affinity than inner defective N, S-C sites, show a comparatively lower output potential of 1.94 V. These results suggest that adjacent C sites near N, S dopants are the most active sites for the Li_2_S_4_ to Li_2_S conversion. From the volcano plot, we found that the potential-limiting step is typically the last step, *LiS to solid Li_2_S(s). This, in turn, indicates that the final conversion governs the overall output potential in the low-potential region, in agreement with previous experimental observations^11^ that the last step exhibits the largest overpotential. It is important to note that in most cases, the reduction of *LiS to Li_2_S serves as the potential-determining step. Given that the adsorption energy of *LiS_x_ radicals scales with the adsorption energy of *LiS, the latter can serve as an effective descriptor for reactions in the low-potential region of the SRR. Moderate *LiS adsorption, as indicated by the Sabatier principle, facilitates the reaction by balancing binding strength and desorption energy, thereby optimizing catalytic performance in the low-potential region.

The computational results highlight the distinct yet complementary roles of Fe sites and N, S-C sites in the SRR. Fe sites exhibit strong adsorption of long-chain polysulfides, such as Li_2_S_8_, enabling efficient activation and reduction in the high-potential region. This is supported by notably higher output potentials on Fe sites compared to solution-based pathways. Additionally, the surface-constrained polysulfides on Fe sites effectively suppress comproportionation-forming Li_2_S_6_ and polysulfide shuttling. On the other hand, N, S-C sites exhibit high activity in the later stages of the SRR, that is, the conversion from Li_2_S_4_ to Li_2_S. Thus, these carbon sites provide moderate adsorption energies for shorter-chain radicals, enabling a thermodynamic driving force for the shift of active sites from Fe sites (*Li_2_S_4_) to carbon sites (*LiS_2_/*LiS_3_), where the vacated Fe site is reoccupied by higher-order polysulfide species (Li_2_S_4_/Li_2_S_8_) for sustained catalytic reduction. This cooperative dual-site synergy ensures that Fe sites dominate the high-potential region while the active carbon sites drive the low-potential processes. The interplay between these dual active sites optimizes the SRR across the entire potential range.

To support the theoretical calculation and demonstrate the cascade catalysis pathway of Fe, N, S-HGF, we further conducted operando Raman studies to quantitatively probe Li_2_S_6_ species in the electrolyte, which is expected to be significantly suppressed when facilitated by the Fe, N, S-HGF cascade catalysis defined in our reaction pathway analysis. Operando Raman spectroscopy of N, S-HGF (Fig. 5d) confirmed the significant presence of Li_2_S_6_ in electrolyte throughout the discharge process (~2.5 V to 2.0 V) (orange line in Fig. 5f), consistent with the previous study^43^. In comparison, operando Raman spectroscopy of Fe, N, S-HGF (Fig. 5e) revealed that the concentration of Li_2_S_6_ formed in Fe, N, S-HGF (red line in Fig. 5f) is substantially lower than that in N, S-HGF, demonstrating the effective role of Fe sites in strongly restraining long-chain polysulfides (Li_2_S_8_ and Li_2_S_4_) on catalyst surface, and thus suppressing comproportionation reaction in electrolyte (Li_2_S_8_ + Li_2_S_4_ → 2Li_2_S_6_), as illustrated in Fig. 1. Together, this operando Raman study provided direct spectroscopy evidence supporting that the presence of Fe sites effectively suppressed the parasitic comproportionation formation of highly soluble Li_2_S_6_ in electrolyte (Li_2_S_8_ + Li_2_S_4_ → 2Li_2_S_6_), further confirming the proposed cascade reaction pathway.

### Performance evaluation of Fe, N, S-HGF-based electrode in practical conditions

**Fig. 6:**
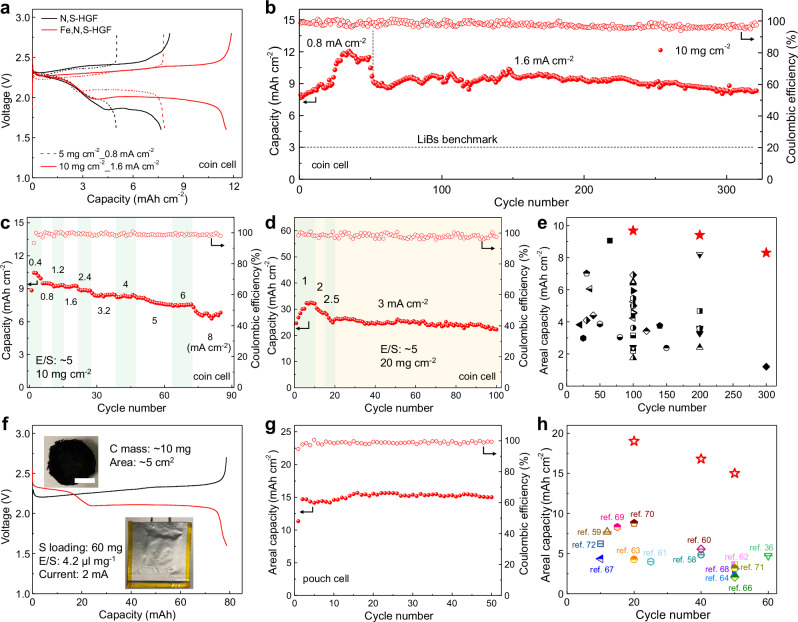
Electrochemical performance of catalytic Fe, N, S-HGF host in Li–S batteries. **a** Charge-discharge curves of N, S-HGF- or Fe, N, S-HGF-based sulfur electrodes at different current densities with a sulfur loading of 5 and 10 mg cm^−2^. **b** Cycling stability of the Fe,N,S-HGF-based sulfur electrodes at the current density of 1.6 mA cm^−2^ with the sulfur loading of 10 mg cm^−2^ (N/P ratio ~20), at a E/S ratio of ~5 µL mg^−1^. **c** Polarization profile of areal capacity response at different current densities. **d** Areal capacities of the Fe, N, S-HGF-based sulfur electrodes with the sulfur loading of 20 mg cm^−2^ and E/S ratio of ~5 µL mg^−1^ at various current densities. **e** The comparison of areal capacity and cycle number among Li-S coin cells, as detailed in Supplementary Table 4. **f** Photo and charge-discharge profile of a 60-mg-sulfur pouch cell with the Fe, N, S-HGF host at a current density of 0.4 mA cm^−2^. Scale bar is 1 cm. **g**, **h** Cycling performance of the assembled pouch cell at a current density of 1 mA cm^−2^ (**g**), and the corresponding comparison of areal capacity versus cycle number between this work and previously reported pouch cells^36,58–72^ (**h**).

Although the electrochemical performance of Li–S batteries has been progressively improved through the use of specifically designed carbon hosts and catalysts, the SRR kinetics and LiPS accumulation and shuttle effect often exacerbate under high sulfur loading (>5 mg cm^−2^) and lean electrolyte (<5 µL mg^−1^) conditions, which are essential for attaining a specific energy density greater than 300 Wh kg^−1^ (Supplementary Fig. 21) in practical devices^51^. To explore the potential of the synergistic Fe, N, S-HGF catalysts in practical conditions (at high mass loading and lean electrolyte conditions), we have further constructed the Li-S coin cell at the sulfur loading of 5 and 10 mg cm^−2^ with a low E/S ratio of 5 µL mg^−1^. The charge-discharge profile revealed that the Fe, N, S-HGF based electrode exhibits a considerably better performance than that of N, S-HGF, showing a notably higher sulfur utilization and lower voltage polarization gap (Fig. 6a). In particular, at 5 and 10 mg cm^−2^, the Fe, N, S-HGF based cells deliver an areal capacity of 7.9 and 11.6 mAh cm^−2^, respectively, realizing a high sulfur utilization of ~94% and ~70% (in comparison with sulfur utilization of ~60% and ~46% for N, S-HGF at 5 and 10 mg cm^−2^). More importantly, the Fe, N, S-HGF-based electrode exhibits highly respectable cycling performance at both 5 and 10 mg cm^−2^ (Fig. 6b, Supplementary Fig. 22), showing a low capacity decay of 0.093% and 0.047% per cycle over 300 cycles, which compares well with the current state-of-the-art catalysts reported for Li-S batteries (Supplementary Table 4). As a result, the coin-cell demonstrates a 320^th^-cycle specific areal capacity of 8.3 mAh cm^−2^, respectively, considerably higher than the typical areal capacity of Li-ion batteries (3 mAh cm^−2^).

We also evaluated the rate capability of Fe, N, S-HGF at 10 mg cm^−2^ (Fig. 6c). Notably, it exhibited a respectable rate capability at the lean electrolyte condition, retaining an areal capacity of ~6.6 mAh cm^−2^ even at a high current density of 8 mA cm^−2^, indicating fast reaction kinetics and Li^+^ transport. At even higher sulfur loading of 20 mg cm^−2^, we achieved a stable areal capacity of 26 mAh cm^−2^ (Fig. 6d). Overall, the areal-capacity performance comparison presented in Fig. 6e clearly highlights the high areal capacity and cycling stability (Supplementary Table 4), which should be originated from the enhanced electrocatalytic activity of Fe, N, S-HGF. On the other hand, when considering the mass overhead of the passive components (e.g., conductive carbon, current collectors ~2.7 mg cm^−2^), the advantage of our HGF electrode becomes more apparent for realizing high gravimetric capacity normalized by the total electrode mass (Supplementary Table 4). With reducing carbon content (<10 wt%), the HGF electrode at 20 mg cm^−2^ (sulfur mass ratio ~91 wt%) can deliver a much higher normalized gravimetric capacity (~1200 mAh g^−1^) than a traditional sulfur electrode (<600 mAh g^−1^) (Supplementary Fig. 23).

We have further scaled up the Fe, N, S-HGF in a pouch cell test (Fig. 6f). With high sulfur loading (12 mg cm^−2^, 85.7 wt% S) and low E/S ratio (4.2 µL mg^−1^), the assembled pouch cell exhibits an areal capacity of 15.8 mAh cm^−2^ ( ~ 79% sulfur utilization), delivering a specific energy density of 442 Wh kg^−1^ (Supplementary Fig. 24). It also shows a stable cycling behavior with a specific capacity of 15 mAh cm^−2^ (Fig. 6g). It is important to note that our Li-S pouch cell with the Fe, N, S-HGF catalytic host delivers substantially higher areal-capacity than some previously reported Li–S pouch cells (Fig. 6h). We also recognize that the laboratory-assembled pouch cell is not optimized, and performance may be further improved with an optimized industry preparation/assembly process.

## Discussion

Using the heteroatom-doped HGF as the model system, we demonstrated that the combination of appropriate metal and non-metal dopants enables synergistic cascade electrocatalysis for multi-step SRR, considerably boosting SRR kinetics and enhancing sulfur utilization in the Li–S chemistry. Our chronoamperometry studies show that the multistep SRR process is kinetically limited by the conversion from Li_2_S_4_ to Li_2_S_2_/Li_2_S, which leads to the accumulation of soluble LiPSs and exacerbates the shuttle effect. We revealed that the Fe, N, S-HGF with dual active sites can help restrain long-chain LiPSs on the surface and facilitate the transfer of intermediates between distinct neighboring active sites, thereby enabling their efficient conversion into the final discharge product. Our operando Raman studies provide direct spectroscopic evidence for the suppressed formation of Li_2_S_6_ during discharge, further confirming the balanced cascade catalysis mechanism. This synergistic electrocatalysis of SRR enables balanced conversion and effectively mitigates the polysulfide shuttle effect. As a result, the Fe, N, S-HGF with dual active sites showed considerably improved performance compared to the N, S-HGF with a single-type active site. At high sulfur loading of 20 mg cm^−2^ with a low E/S ratio of 5 μL mg^−1^, the Fe, N, S-HGF-based cell obtained stable cycling with the high areal capacity of ~26 mAh cm^−2^. The lab-assembled Li-S pouch cell also exhibited respectable cycling stability with a specific energy density of 442 Wh kg^−1^, demonstrating its potential in practical conditions. Our findings establish a theoretically mapped and experimentally validated cascade pathway for SRR catalysis, elucidating its essential role in driving balanced LiPS conversion, kinetically suppressing LiPS accumulation and shuttle. The principles demonstrated here can be extended to other electrocatalyst systems, providing a framework for the rational design of future electrode materials for Li-S batteries and beyond.

## Methods

### Synthesis of heteroatom-doped holey graphene framework

Graphene oxide was prepared similarly via our previous method. 6 g natural graphite flakes were added to 140 ml concentrated sulfuric acid under vigorous stirring in the ice-water bath, and then, 3 g sodium nitrate (Sigma-Aldrich) and 18 g potassium permanganate (Sigma-Aldrich) were added slowly and carefully to avoid potential accidents. Considering the strong acidity of sulfuric acid and the strong oxidizing properties of sodium nitrate and potassium permanganate, it is important to keep the temperature near 0 °C to realize a slow oxidation of the graphite. After stirring for 30 min, the reaction mixture was transferred to a water bath at 50 °C to form a thick paste under stirring. Subsequently, it was transferred back to the ice-water bath, and 1 L iced deionized water was added dropwise. The mixture was centrifuged and washed three times by using a 1:10 HCl aqueous solution, followed by repeated washing with deionized water. The final solution was dialyzed for about 7 days to remove the extra ions absorbed on the graphene oxide surfaces.

A typical hydrothermal process^52^ was applied to form the HGF morphology with interconnected micro- and nanopores. Heteroatom-doped HGFs were synthesized by reacting the dopant sources with the holey graphene oxide (HGO) aqueous dispersion through a typical hydrothermal method. Aqueous HGO solution was synthesized by mixing 50 ml of 2 mg ml^−1^ GO solution with 5 ml of 30% H_2_O_2_ at 100 °C under stirring for 2 h. Specifically, 1.5 g of NH_4_SCN powders were added to 20 ml 2 mg ml^−1^ HGO solution, followed by mixing and sonication to obtain a homogeneous mixture. The hydrothermal treatment was then conducted at 180 °C for 6 h to produce a free-standing N, S-HGF hydrogel. To form the aerogel, the hydrogel was freeze-dried and annealed in Ar at 900 °C for 1 h. The metal-doped samples (e.g., Fe, N, S-HGF, Co, N, S-HGF, or Ni, N, S-HGF) were synthesized by a second-step hydrothermal process. The as-prepared N, S-HGF hydrogel was immersed into 1 ml H_2_O, with the addition of 15–20 µl 3 mg ml^−1^ metal chloride solutions (e.g., FeCl_3_ (98%, Alfa Aesar), CoCl_2_ (97%, Sigma-Alderich), or NiCl_2_ (ReagentPlus, Sigma-Alderich)) for 48 h, and then washed and immersed into 1 ml 0.015 mg ml^−1^ metal chloride solutions, followed by the hydrothermal reaction at 180 °C for 6 h in the autoclave. The control sample, Fe, N-HGF, was synthesized by changing the dopant source to FeCl_3_ and NH_4_Cl and following the same procedures as N, S-HGF.

### Preparation of electrolyte and polysulfide solutions

The electrolyte (denoted as blank electrolyte) was prepared via dissolving 1 M lithium bis(trifluoromethanesulfonyl) imide (99.99%, Sigma-Aldrich) and 0.2 M lithium nitrate (ReagentPlus, Sigma-Aldrich) into the mixed 1,2-dimethoxyethane (99.5%, anhydrous, Sigma-Aldrich) and 1,2-dioxolane (99.8%, anhydrous, Sigma-Aldrich) solution (1:1 by volume). Note that the solvent was treated with the molecular sieve before the electrolyte preparation. The Li_2_S_6_ catholyte was prepared by reacting the sublimed sulfur (99.98%, Sigma-Aldrich) with Li_2_S (99.98%, Sigma-Aldrich) in stoichiometric proportions in the blank electrolyte. The mixture was vigorously stirred at 50 °C in an argon-filled glove box overnight to produce a brown-red Li_2_S_6_ solution.

### Electrochemical measurements

The electrocatalytic SRR activity was tested by the RDE technique in an argon-filled glovebox (O_2_ < 1 ppm, H_2_O < 1 ppm) with a CHI 760E electrochemical workstation. A catalyst ink (2 mg ml^−1^) was first prepared by sonicating 2 mg of catalysts in 1 ml ethanol with 20 μl 5 wt% Nafion solution, and 10 μl catalyst ink was then drop-cast onto a glassy carbon electrode (0.196 cm^2^) to form a flat film electrode with a mass loading of 0.1 mg cm^−2^. The RDE test was performed in a two-electrode open-cell by using a lithium foil as the counter and reference electrode and a catalyst electrode as the working electrode. The electrolyte solution used for RDE tests was 4 mM sulfur dissolved in the blank electrolyte. Before the RDE test, the catalyst film electrode was first activated in the blank electrolyte by scanning the cyclic voltammetry (CV) for 50 cycles at 10 mV s^−1^ in the range of 3.1 V to 3.0 V. Linear sweep voltammetry measurements were then conducted in the 4 mM sulfur solution at a scan rate of 20 mV s^−1^ in the range of 3.3 V to 1 V.

The electrochemical performance was conducted in the CR2032 coin cells assembled in an argon-filled glovebox. The electrode was prepared by directly compressing the aerogel into a freestanding thin film, and the mass of the thin film can be controlled by tuning the height of the aerogel. Next, Li_2_S_6_ catholyte as the sulfur source was directly added into the catalyst electrode by a drop-casting method. The sulfur electrodes were then directly assembled into a CR2032 coin cell with a lithium chip (15.6 mm × 0.45 mm), Celgard 2500 separator (Shenzhen Tianchenghe Technology Co., Ltd.) and blank electrolyte (E:S ratio = 20 μl mg^−1^). In the coin cell test with low mass loading (1 mg cm^−2^ of sulfur), the low mass ratio of sulfur (33%) in the positive electrode and high E/S ratio can ensure complete conversion from S_8_ to Li_2_S and mitigate the influence from other factors (e.g., Li^+^ diffusion or electron transport) for the direct comparison of SRR catalytic activity. For the test at high mass loading (5–20 mg cm^−2^), the mass ratio of sulfur in the positive electrode is ~75–91 wt%, and the electrolyte/sulfur ratio is set to be 5 µl mg^−1^ unless otherwise specified. The galvanostatic tests were performed in the voltage range of 1.6 V–2.8 V at various C rates (1 C = 1675 mAh g^−1^). The calculation of the specific capacity of coin cells is based on the mass of sulfur active material, and the areal capacity is based on the geometric area of the positive electrode. No climatic/environmental chamber is used for electrochemical tests.

Typically, for the Fe,N,S-HGF/sulfur electrode used for the pouch cell test, the large-size aerogel was prepared via a similar process and compressed into the free-standing electrode (area: ~5 cm^2^, mass: ~10 mg). Similarly, the Li_2_S_6_ catholyte (sulfur mass: ~60 mg) was drop cast onto the aerogel electrode to obtain the sulfur electrode. The pouch cell assembly process was carried out in an argon-filled glovebox by assembling the sulfur electrode, the lithium foil and the Celgard polypropylene separator (~25 µm) into Al-plastic films, followed by the injection of the blank electrolyte (E/S ratio = 4.2 μl mg^−1^) and final encapsulation in an argon-filled glovebox. The Al and Ni tabs were attached with the positive and negative electrodes, respectively, and introduced for outward connection. Electrochemical measurements of pouch cells were conducted similarly, in which the external pressure was applied using two glass slides and clamps. The detailed evaluation of specific energy density can be found in Supplementary Fig. 24.

CV curves of coin cells were recorded in the voltage range from 1.6 to 2.8 V at a scan rate of 0.05 mV s^−1^. A quantitative charge analysis of CV curves was conducted to evaluate the different stages in the SRR via the integration of the peak area in different potential regions. The electron number transferred (*Q*) for the electrochemical steps in the SRR can be calculated from CV curves. *Q* is calculated by integrating the area enclosed in CV:

1 $$Q=\int^{{t}_{2}}_{{t}_{1}}i\left(t\right){dt}=\frac{1}{v}\int^{{V}_{2}}_{{V}_{1}}i\left(V\right){dV}=\frac{A}{v}\int^{{V}_{2}}_{{V}_{1}}j\left(V\right){dV}=\frac{{AS}}{v}$$

where *Q* is charge (C), *i* is current intensity (A), *j* is current density (A cm^−2^), *A* is the geometric area of the electrode (cm^2^), *v* is scan rate (V s^−1^), *t* is time (s), *V* is voltage (V) and *S* is integrated area.

### Material characterizations

The morphology and structure of the resulting materials were characterized by SEM (Zeiss Supra 40VP) and XPS (Kratos AXIS Ultra DLD spectrometer). Annular dark field scanning transmission electron microscopy (ADF-STEM) was performed on a JEOL GrandARM300CF (JEOL Ltd., Japan), located in the electron Physical Sciences Imaging Centre (ePSIC) at Diamond Light Source, operating at 80 kV accelerating voltage, with a convergence semi-angle of 32 mrad (46.5 pA beam current) and ADF collection inner and outer semi-angles of 68-206 mrad, respectively. Data were processed with the Gatan Microscopy Suite (“Digital Micrograph”; Gatan Inc., USA). A low-pass filter has been used to improve the visibility of dopants and the graphene lattice and aid qualitative intensity analysis. The outer bound of the filter was set to the frequency of the 1st order graphene reflections, with an exponential taper added to the filter’s edge to minimize the introduction of edge artifacts while still overall reducing high frequency noise components.

### XAS characterizations

X-ray absorption spectroscopy (XAS) measurements were carried out at beamline 4–1 of the Stanford Synchrotron Radiation Lightsource (SSRL), utilizing the Fe K-edge in a fluorescence geometry with a 30-element Canberra germanium detector. For energy calibration, an Fe reference foil was positioned after the sample holder and collected simultaneously. XAS data were collected up to a wavenumber (k) of 12.1 Å^−1^. The data were processed and analyzed using ATHENA and ARTEMIS, tools within the IFEFFIT software suite^53^. Spectra were calibrated, normalized, and background-subtracted according to the protocols detailed in ATHENA’s documentation. We attempted to fit the EXAFS data with scattering paths based on the proposed model (Fig. 2f). However, accurate fits were not achieved due to the number of free variables and the challenges associated with fitting similar scattering paths of light elements (C, N, or O) under the broad first nearest neighbor peak. Nevertheless, models with an S atom nearest neighbor resulted in nonphysical coordination numbers, supporting our conclusion that the Fe and S are not coordinated.

### Operando Raman spectroscopy

Raman spectroscopy measurements were collected using a Horiba XploRA+ confocal Raman microscope with the ×10 objective. The sample was subject to a 532 nm laser for 2 s and averaged 100 times. Data were collected with a 2400 cm^−1^ grating and 50 µm slit. For operando Raman spectroscopy, a regular coin cell was modified with a transparent window on the positive electrode side to allow laser light to enter. The laser was focused on the electrolyte near the boundary between the heteroatom-doped HGF and the electrolyte. The Raman cell was run with a discharge CV scan at 0.05 mV s^−1^ when data were being collected. The resulting Raman spectra were corrected by subtracting the blank electrolyte peaks and baseline, and subsequently, we conducted the quantitative analysis of polysulfides. The peak at around 399 cm^−1^ was used for the quantification of Li_2_S_6_. More details can be found in our previous work^43^.

### DFT calculations

All the calculations are performed with DFT using the Vienna ab initio simulation package^54^. The strongly constrained and appropriately normed functional is used^55^. Gaussian smearing with a sigma value of 0.1 is used. All calculations for molecules, surface, and surface with adsorbates are spin-polarized with a cutoff energy as 500 eV. 1 × 1 × 1, 1 × 3 × 1, and 3 × 1 × 1 k-point meshes are used for terrace, armchair, and zigzag models, respectively. A Γ point only k-point mesh is used for molecules. The solvation effects are described using a hybrid model, which is noted as micro-solvation: the first solvation shell is described using explicit solvation molecules, and the rest is described by an implicit model. DOL molecule is chosen to describe the first solvation shell since the dielectric constant, 7.13, is close to that of the mixtures. The implicit solvation is described using the VASPSol add-on package, which implements the linearized Poisson-Boltzmann model in the periodic boundary condition^56^. The dielectric constant is set as 7.0. We use a cutoff charge, as 0.0002 Å^−3^ to avoid the unphysical invasion of the implicit solvent invasion into the first solvation shell. Benchmarks of functionals and explicit solvent are seen in our previous work^43^. Optimized structures can be found in Supplementary Data.

We adopted half of the gas-phase entropy to estimate the entropy of aqueous phase DOL. All the vibrational components are calculated within the harmonic approximation. For each of these close shell Li_2_S_x_ species, we consider each Li is solvated by a first shell of 3DOL molecules, ring-like polysulfides are considered in this work since they are more stable than chain-like structures. The Li_2_S_x_ part and the 6DOL part are treated separately: the vibrational modes on the Li_2_S_x_ part of each model are calculated, and the contribution of the vibrational modes on the DOL molecule part is approximated to be the same as the DOL contribution in 6DOL-Li_2_S_4_.

When considering the chemical potential of the Li^+^ cation and electron pair, we treat the potential effects by analogy with the computational hydrogen electrode (CHE)^57^: the chemical potential of the Li^+^ cation and an electron is linked with the bulk Li via:

2 $$G({{{{\rm{Li}}}}}^{+}+{{{{\rm{e}}}}}^{-})=G({{{{\rm{Li}}}}}_{{{{\rm{bulk}}}}})-{{{{\rm{e}}}}U}_{{{{\rm{Li}}}}/{{{\rm{Li}}}}+}-({{\mathrm{ln}}}10){k}_{{{{\rm{b}}}}}{{{\rm{TpLi}}}}$$

where *U* is the potential versus Li/Li^+^ electrode and pLi is the negative base 10 logarithm of the Li^+^ concentration in the solution. The experimental Li^+^ concentration is around 1 M, and the *k*_*b*_*TpLi* term vanishes. The Gibbs free energy becomes:

3 $$G({{{{\rm{Li}}}}}^{+}+{{{{\rm{e}}}}}^{-})=G({{{{\rm{Li}}}}}_{{{{\rm{bulk}}}}})-{{{{\rm{e}}}}U}_{{{{\rm{Li}}}}/{{{\rm{Li}}}}+}$$

The output potential (*U*) is directly linked with the reaction free energy(*G*_*rxn*_):

4 $$U=-{\Delta G}_{{{{\rm{rxn}}}}}/{{{\rm{nF}}}}$$

where ∆*G* is the reaction free energy, *n* is the number of electrons involved in the reaction and *F* is the Faraday’s constant.

## Supplementary information

Supplementary Information

Description of Additional Supplementary Files

Supplementary Data

Transparent Peer Review file

## Source data

Source Data

## Data Availability

The data that support the findings of this study are available in the main text, figures and Supplementary Information files. Source Data file has been deposited in Figshare under accession code 10.6084/m9.figshare.31937169. Source data are provided with this paper.
