# Supplementary material for: Designing and mapping cascade catalysis pathway for balanced polysulfide conversion in Li-S batteries
Source: Nat Commun. 2026 Jul 1;17:8191. doi: 10.1038/s41467-026-74160-3 (PMC13462536; doi:10.1038/s41467-026-74160-3)
Supplement: Supplementary file 1 [file 41467_2026_74160_MOESM1_ESM.pdf]

Designing and mapping cascade catalysis pathway for balanced polysulfide conversion in Li-S batteries

Leyuan Zhang[1,2,8], Dongfang Cheng[3,8], Pu Zhang[4], David G. Hopkinson[5], Zhaozong Wang[1], Ao Zhang[2], Chen Li[1], Ran Wang[2], Rongli Liu[1], Christopher S. Allen[5,6], Johanna Nelson Weker[4], Yu Huang[2,7], Philippe Sautet[1,3]*, Xiangfeng Duan[1,7]*

This file contains:

Supplementary Figure 1-24

Supplementary Table 1-4

References 1-45|

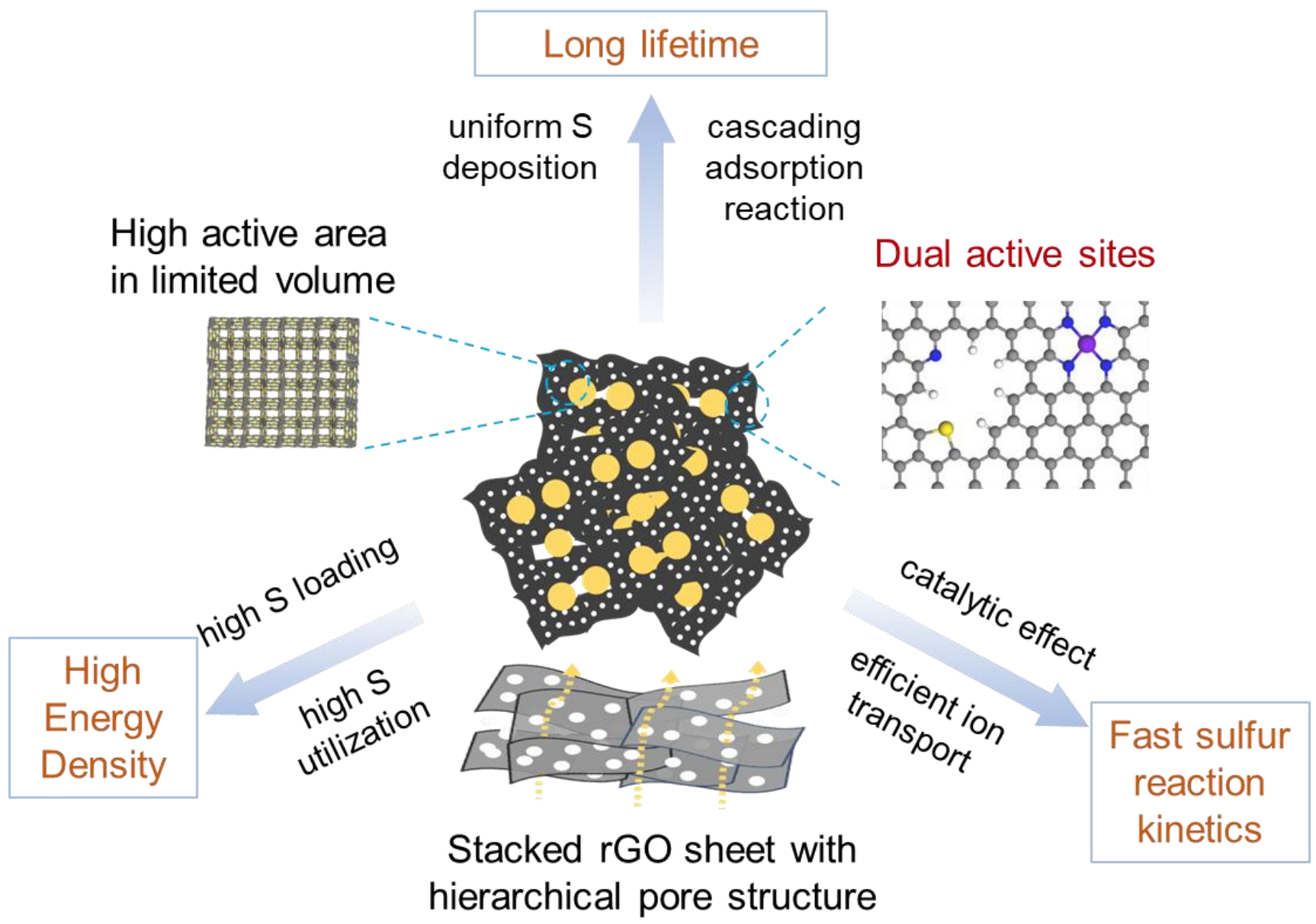

Supplementary Figure 1 | Design strategy and advantageous features of heteroatom-doped HGF host for sulfur reduction chemistry. The atomic doping site can have strong interaction with polysulfide species and Li^+ to mitigate the shuttle effect and facilitate the sulfur reduction reaction via the catalytic effect, leading to fast sulfur reaction kinetics and high sulfur utilization. The hierarchical pore structure of HGF host with stacked holey graphene sheet can accommodate both high sulfur loading (high energy density) and fast lithium-ion transport, which is beneficial to demonstrating high-energy and high-power Li-S chemistries. The sufficient triple-phase (electrolyte/Li_2S/dual catalytic sites) associated with synergistic cascade catalysis, high active area, and confined reaction space, can contribute to the uniform and reversible Li_2S deposition, which is beneficial for long-term cycling stability.

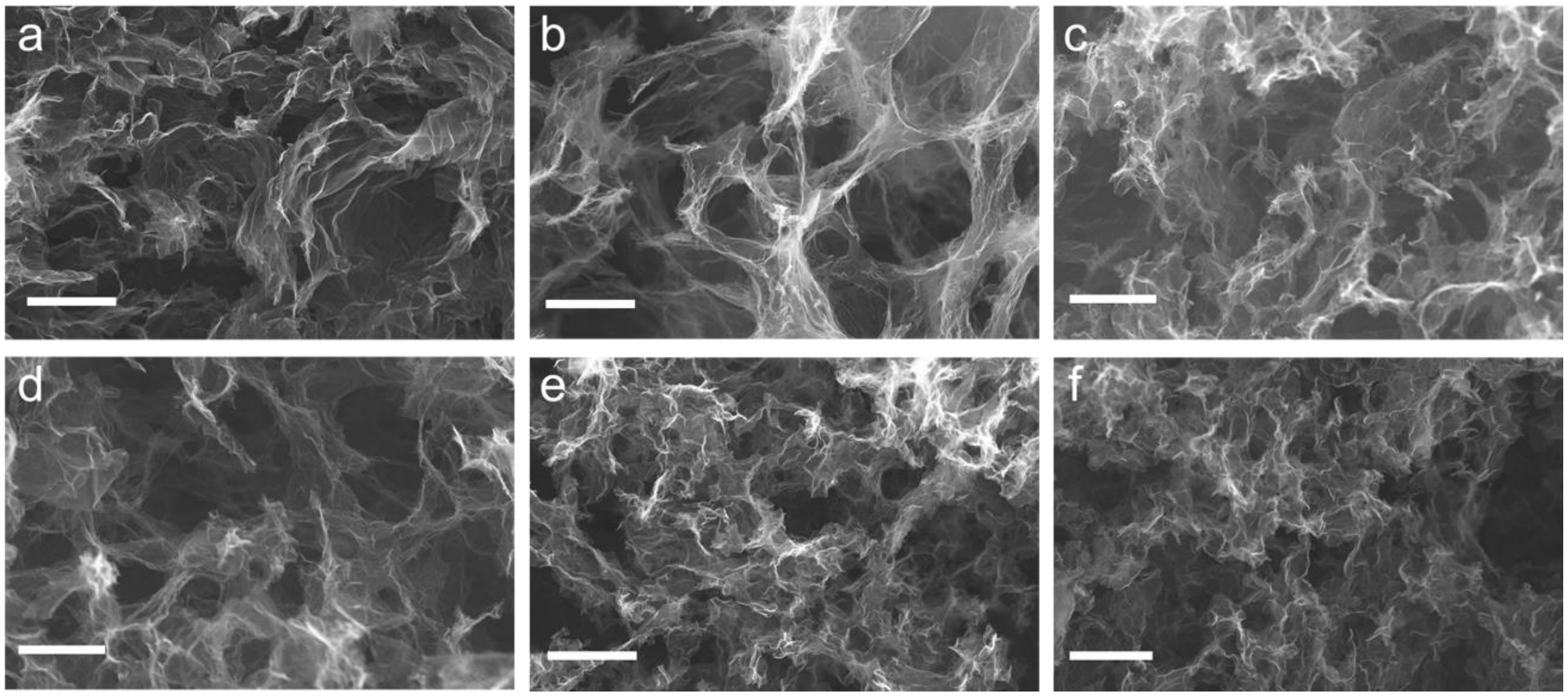

Supplementary Figure 2 | SEM images of different heteroatom-doped HGFs. a, HGF; **b**, N,S-HGF; **c**, Fe,N-HGF; **d**, Fe,N,S-HGF. **e**, Co,N,S-HGF; **f**, Ni,N,S-HGF. All heteroatom-doped HGFs show similar hierarchical porous structures. Scale bars: 5 μm.

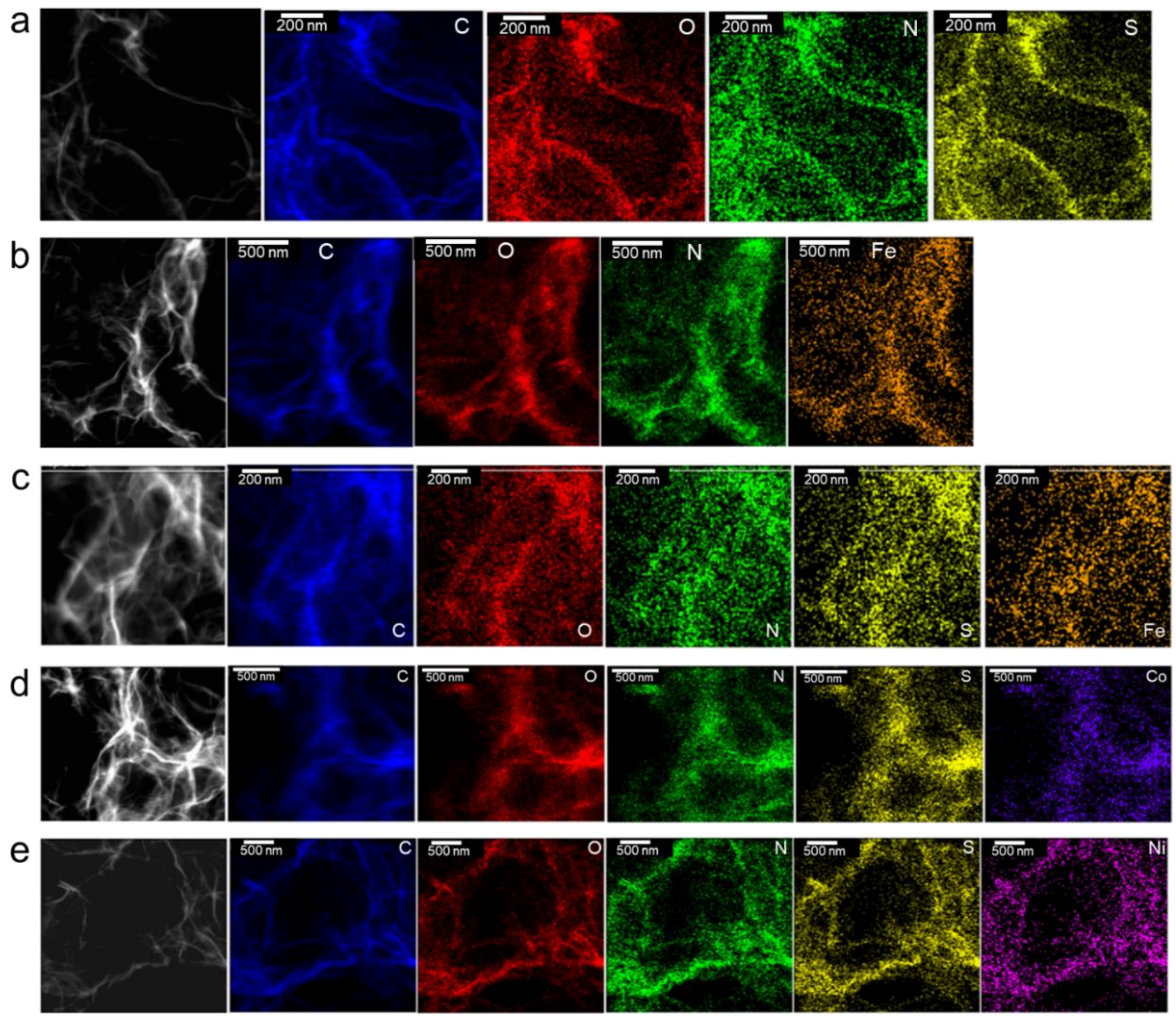

Supplementary Figure 3 | Elemental distribution of heteroatom-doped HGFs. **a-e**, TEM EDS mapping of N,S-HGF (**a**), Fe,N-HGF (**b**), Fe,N,S-HGF (**c**), Co,N,S-HGF (**d**), and Ni,N,S-HGF (**e**).

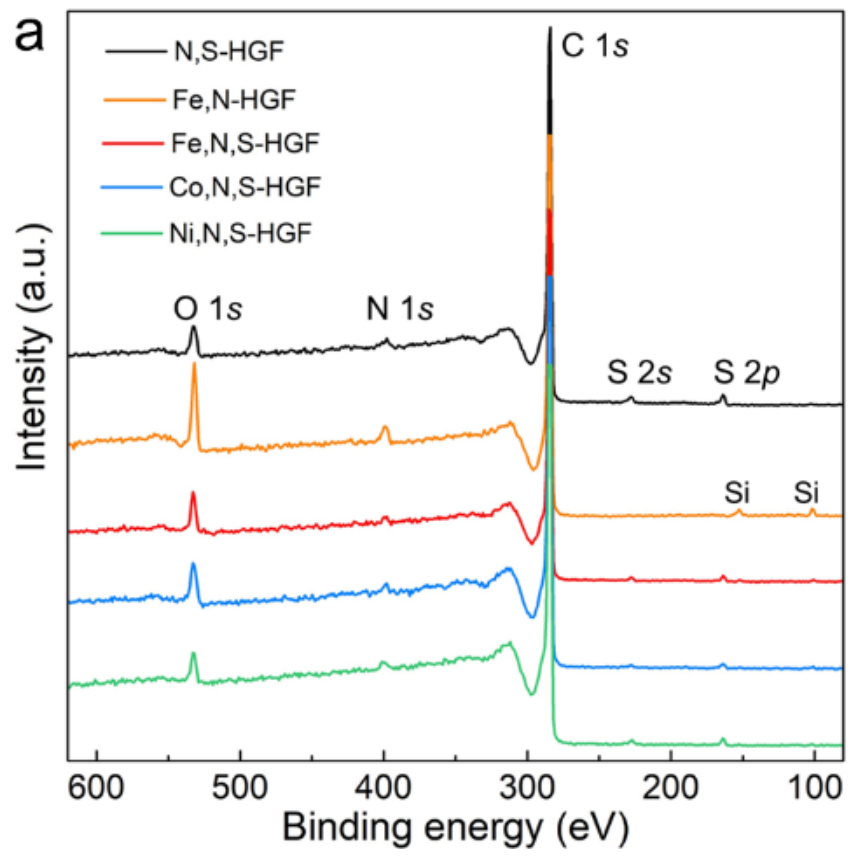

b

at%	C	O	N	S	metal
N,S	93.30	3.90	1.51	1.28	/
Fe,N	90.34	7.07	2.57	/	/
Fe,N,S	93.98	4.10	1.34	0.57	/
Co,N,S	94.59	3.65	1.36	0.41	/
Ni,N,S	95.81	2.44	1.16	0.61	/

Supplementary Figure 4 | XPS spectra of heteroatom-doped HGFs. **a**, XPS survey spectra of various HGFs shows the distinct peaks of N (~400 eV) or S (~164.8 and 228.5 eV) signals, suggesting the doping of N or S elements. While for the metal heteroatoms (i.e, Fe, Co, or Ni), they are hardly detected in the XPS characterization, indicating a low concentration (≤ 0.1 at%). (a.u. represents arbitrary units) **b**, A summary of atomic ratios of C, O, N, S, and metal elements in heteroatom-doped HGFs.

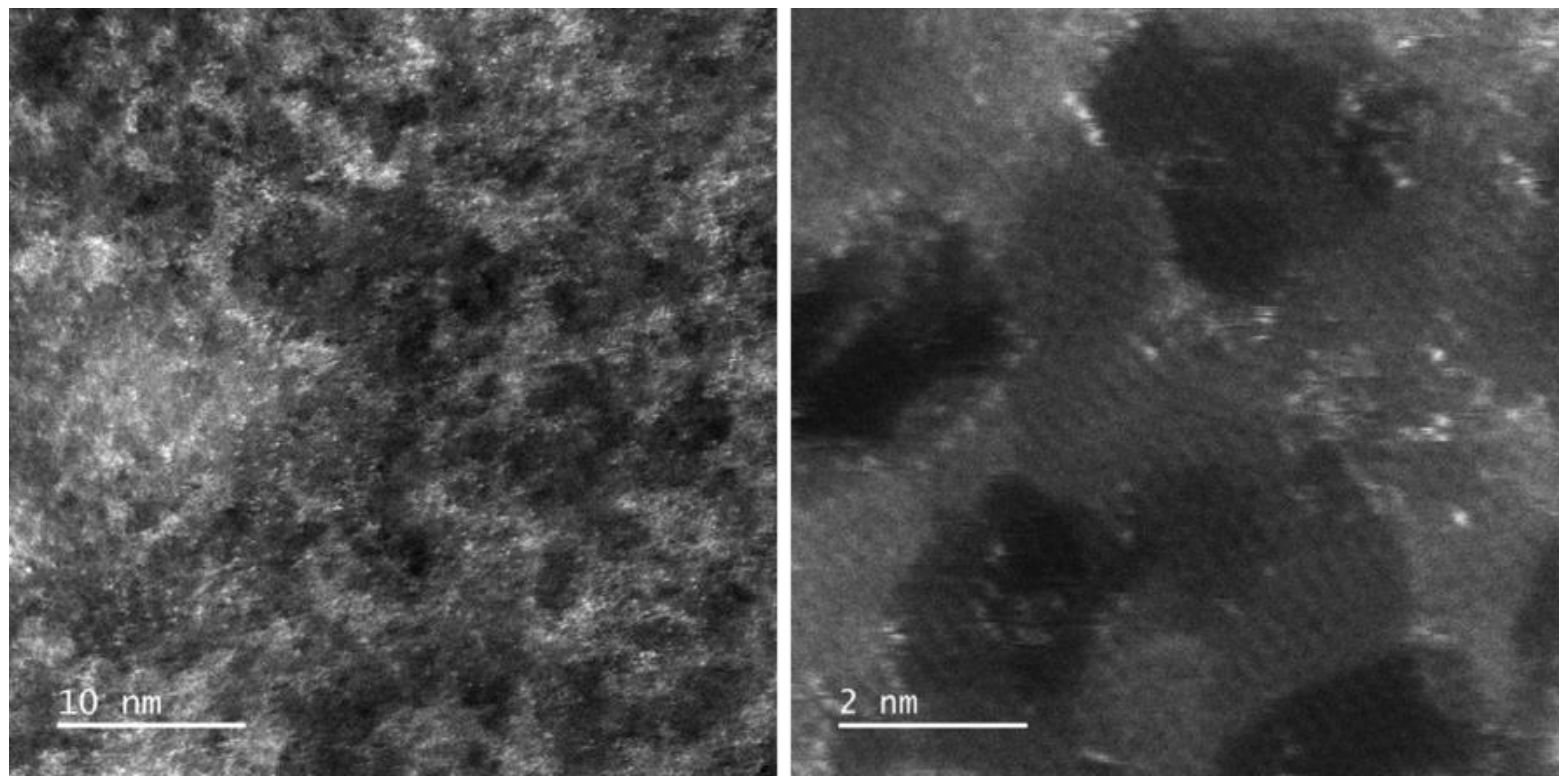

Supplementary Figure 5 | Annular dark-field scanning transmission electron microscopy (ADF-STEM) images of Fe,N,S-HGF. It shows the pores and Fe and S dopants in the graphene matrix.

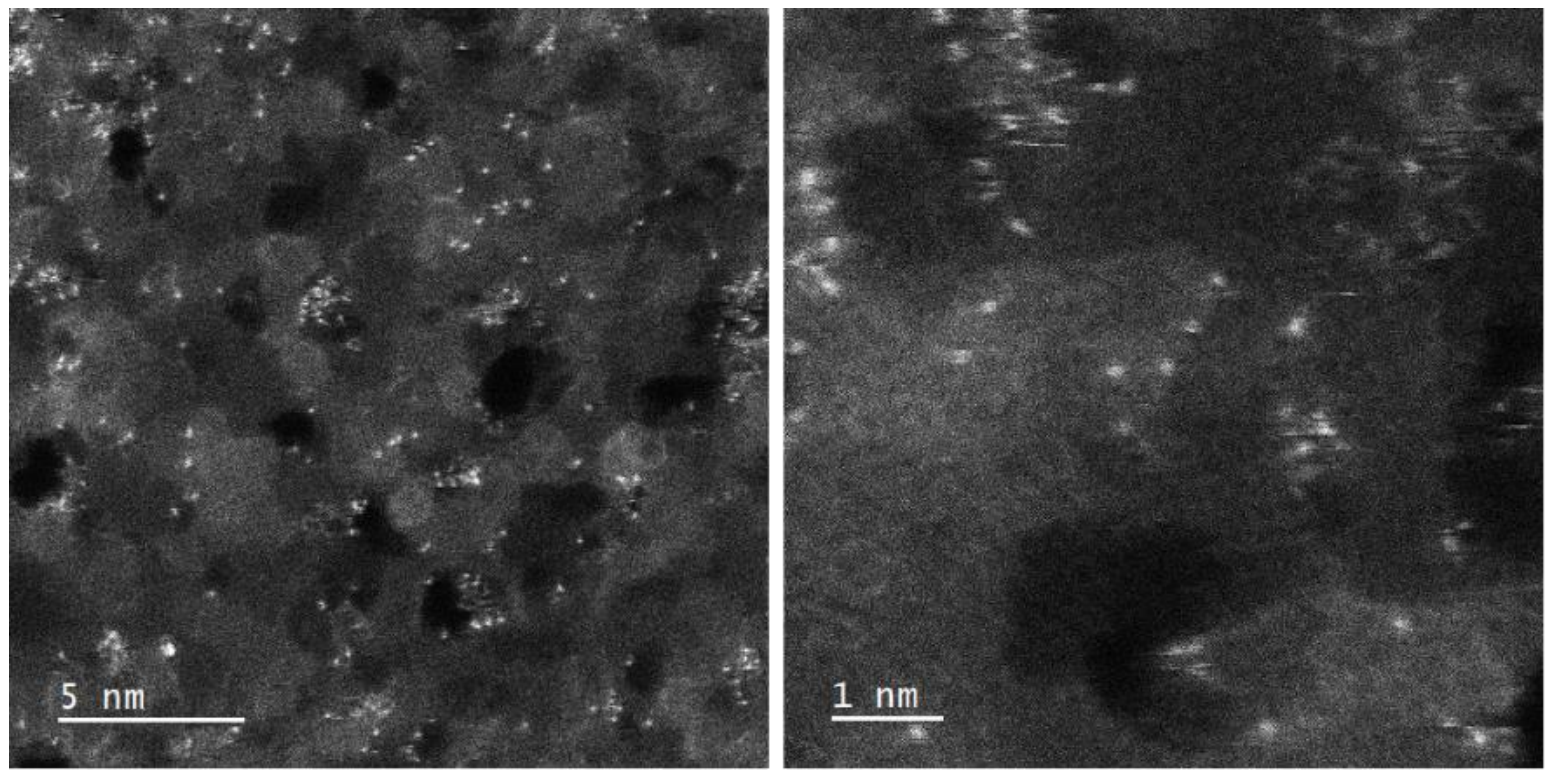

Supplementary Figure 6 | Annular dark-field scanning transmission electron microscopy (ADF-STEM) images of Fe,N-HGF. It shows the pores and Fe dopants in the graphene matrix.

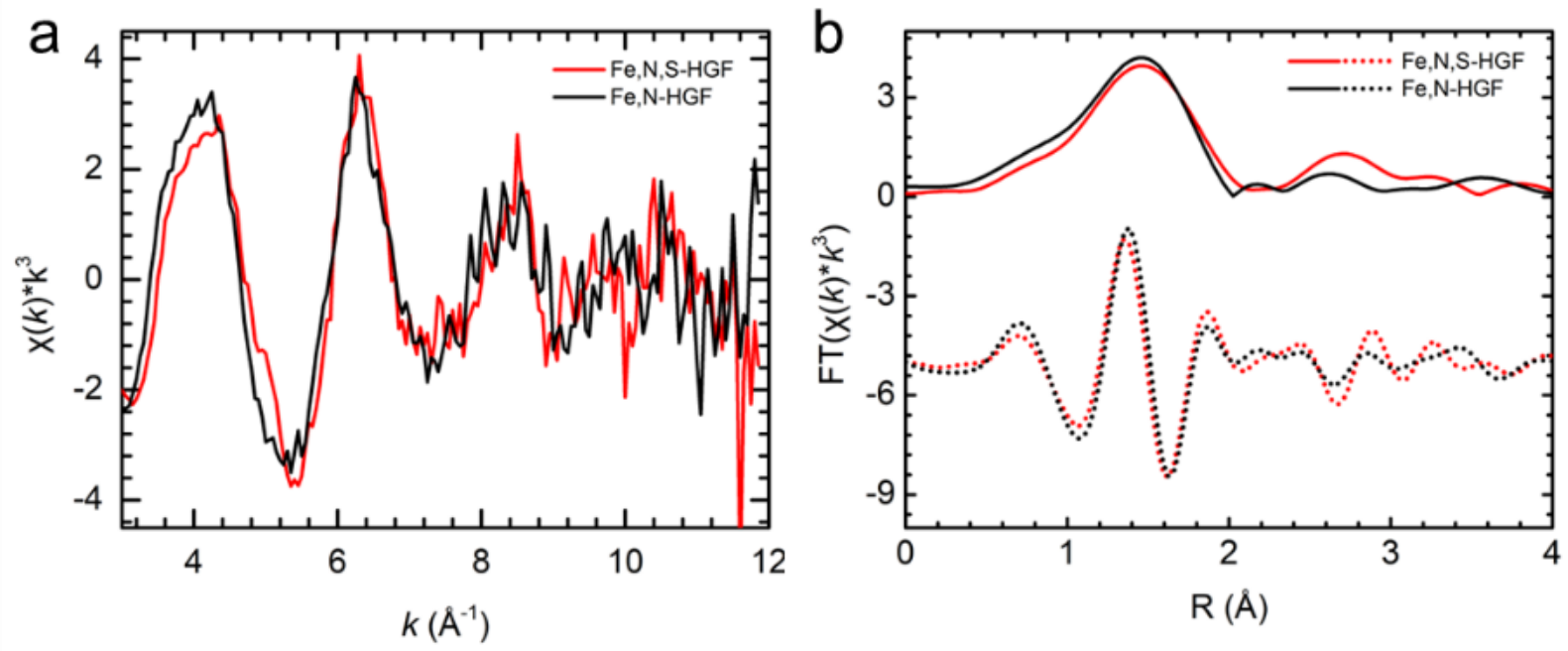

Supplementary Figure 7 | The comparison of Fe K-edge EXAFS for Fe,N,S-HGF and Fe,N-HGF. a, Fe K-edge EXAFS for Fe,N,S-HGF (red) and Fe,N-HGF (black), shown in k^3-weighted *k*-space. **b**, Fe K-edge EXAFS for Fe,N,S-HGF (red) and Fe,N-HGF (black), shown in R-space (FT magnitude and imaginary component). The data are k^3-weighted and not phase corrected.

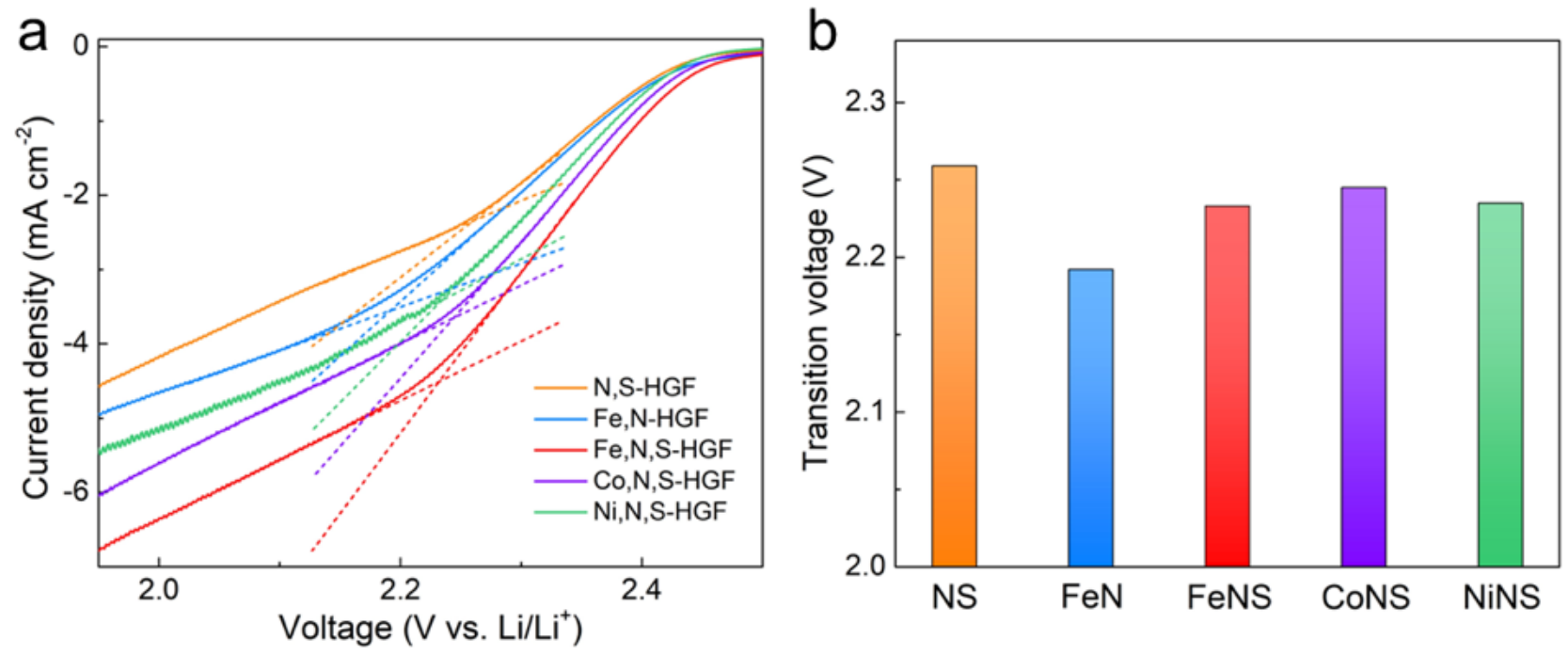

Supplementary Figure 8 | The transition voltage in the LSV curves of various HGFs. **a**, The enlarged LSV curves of heteroatom-doped HGFs with the applied tangent lines. **b**, The transition voltage determined in the LSV curves via the tangent lines. Based on the tangent line of the LSV curves, the resulting transition voltages revealed that all catalysts with N,S-C sites exhibit transition points at higher potentials than that without N,S-C sites: N,S-HGF(2.26 V) > Co,N,S-HGF (2.25 V) > Ni,N,S-HGF (2.24 V) > Fe,N,S-HGF (2.23 V) > Fe,N-HGF (2.19 V). The higher transition voltage suggests higher onset potentials and more favorable kinetics for the $Li_2S_4 \rightarrow Li_2S$ conversion when there are N,S-C sites, demonstrating the critical role of N,S-C sites for the 2nd-step conversion.

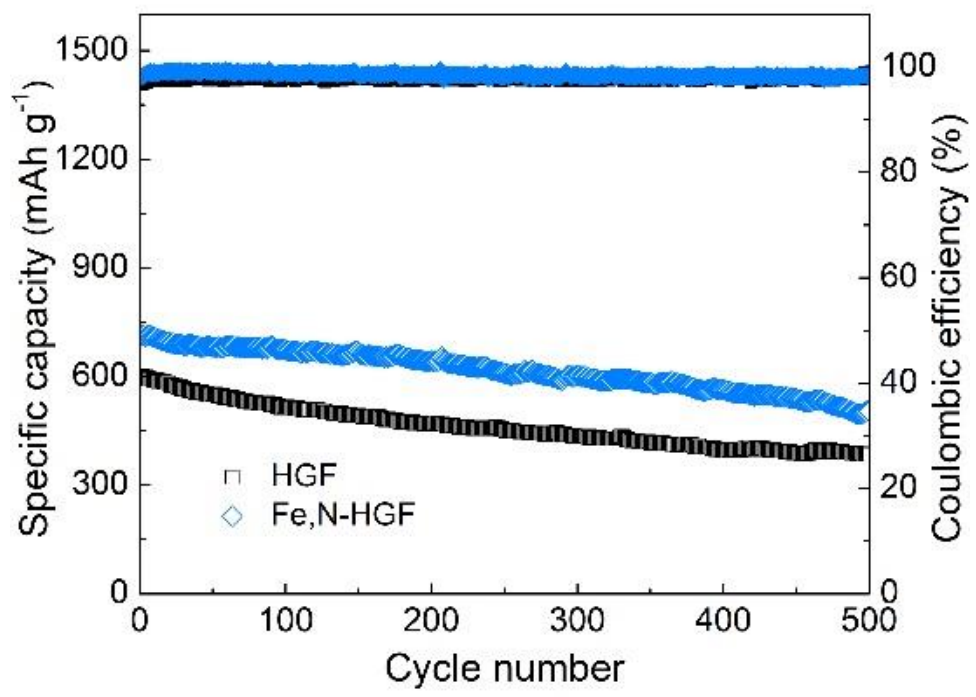

Supplementary Figure 9 | Cycling performance of HGF and Fe,N-HGF based electrodes at a current density of 1 C.

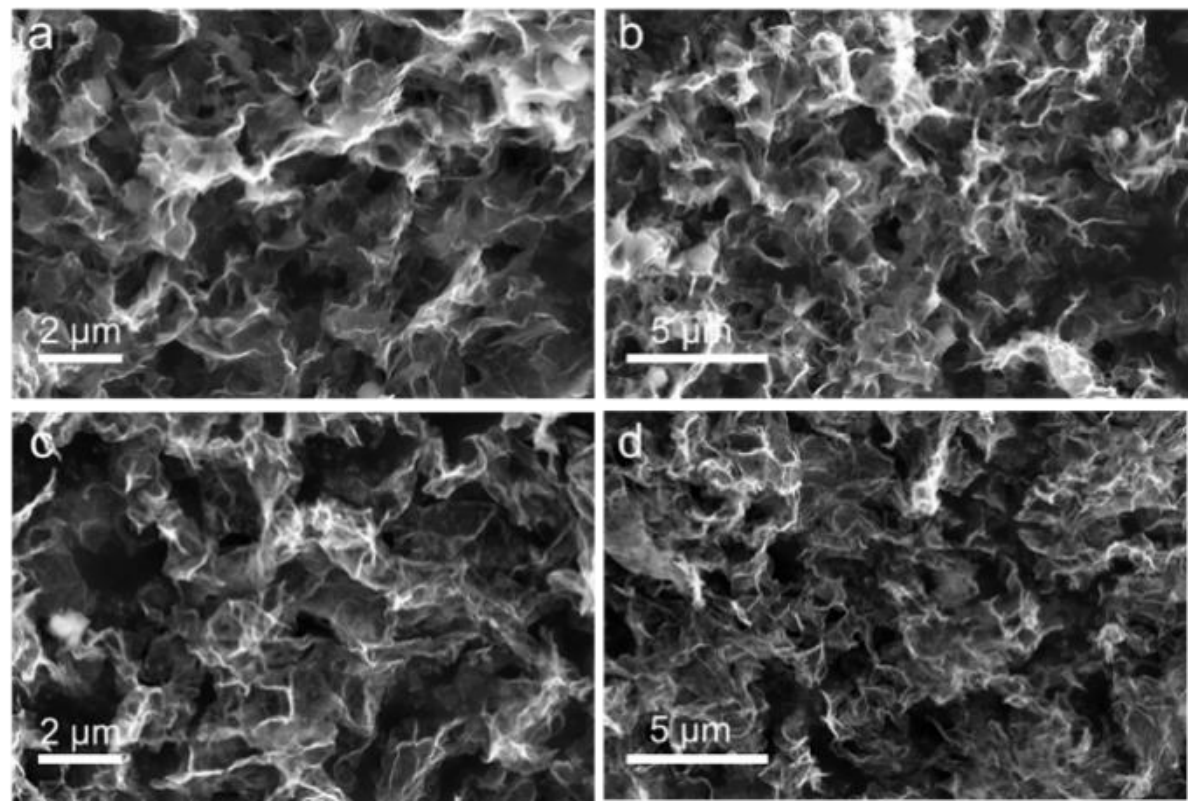

Supplementary Figure 10 | SEM characterization of post-cycling N,S-HGF electrodes. a,**b**, SEM images of the N,S-HGF electrode cycled for 10 cycles. **c**,**d**, SEM images of the N,S-HGF electrode cycled for 500 cycles.

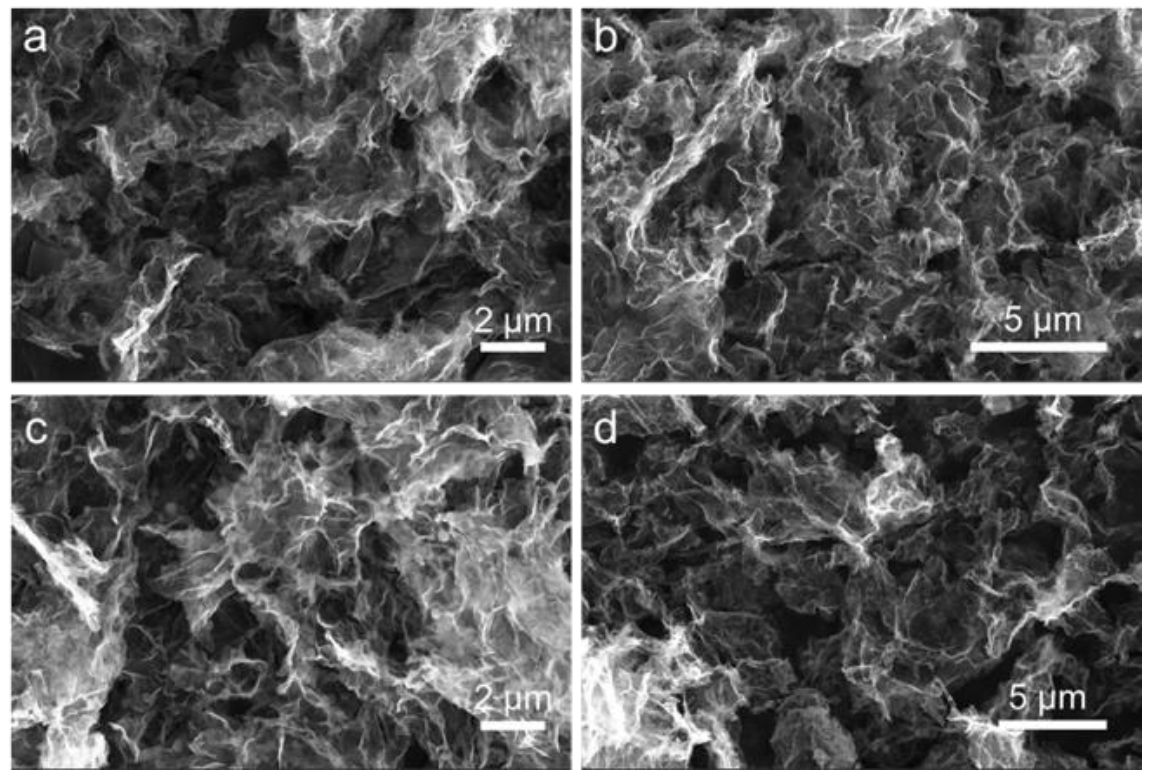

Supplementary Figure 11 | SEM characterization of post-cycling Fe,N,S-HGF electrodes. a,**b**, SEM images of the Fe,N,S-HGF electrode cycled for 10 cycles. **c**,**d**, SEM images of the Fe,N,S-HGF electrode cycled for 500 cycles.

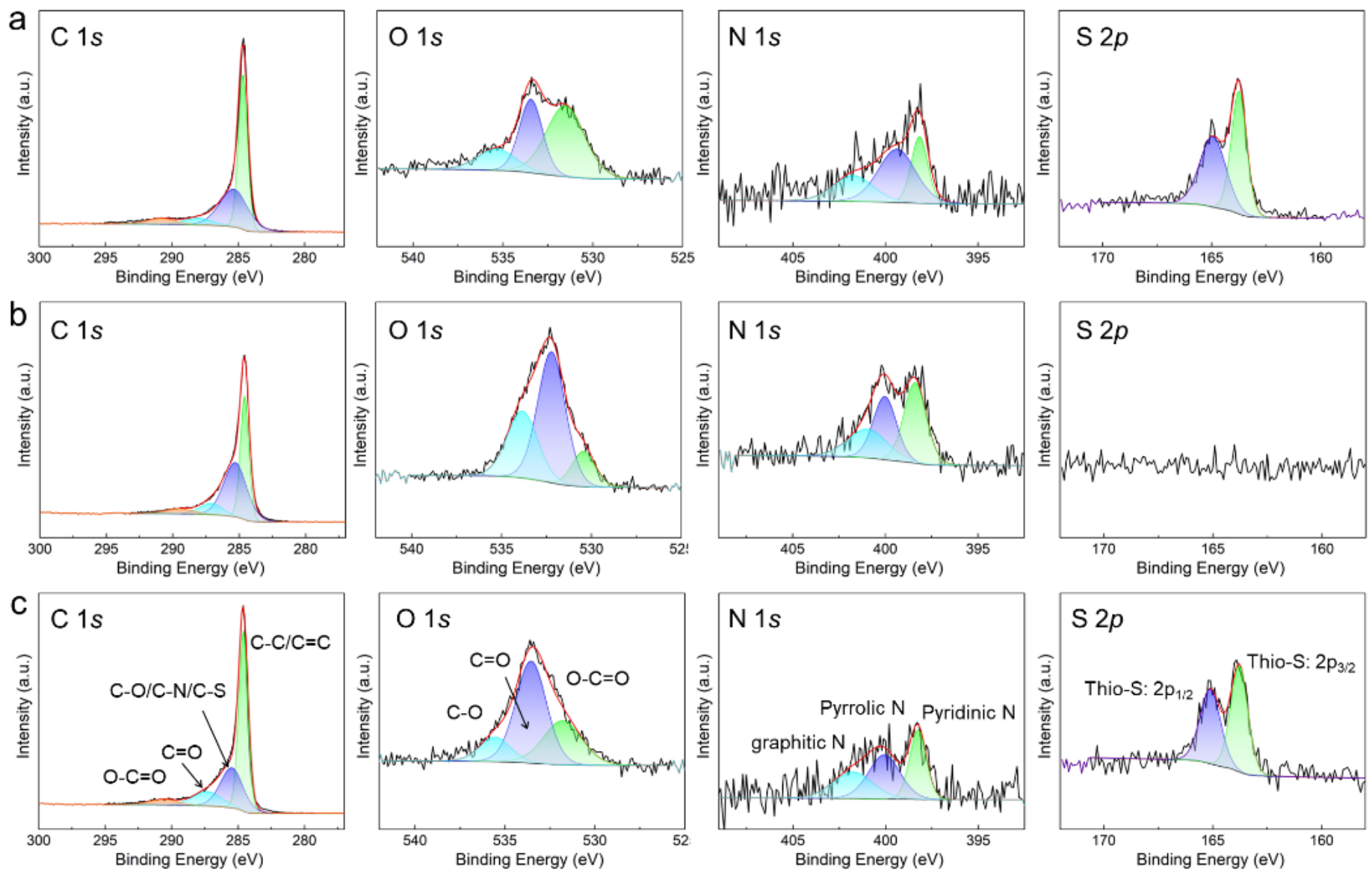

Supplementary Figure 12 | XPS analysis of various HGFs. High-resolution XPS spectra of pristine N,S-HGF (**a**), Fe,N-HGF (**b**), Fe,N,S-HGF (**c**). C 1*s*, O 1*s*, N 1*s*, and S 2*p* spectra are presented, including peak deconvolution and assignments. It reveals that the bond environments for N or S dopants are similar in pristine N,S-HGF and Fe,N,S-HGF. (a.u. represents arbitrary units)

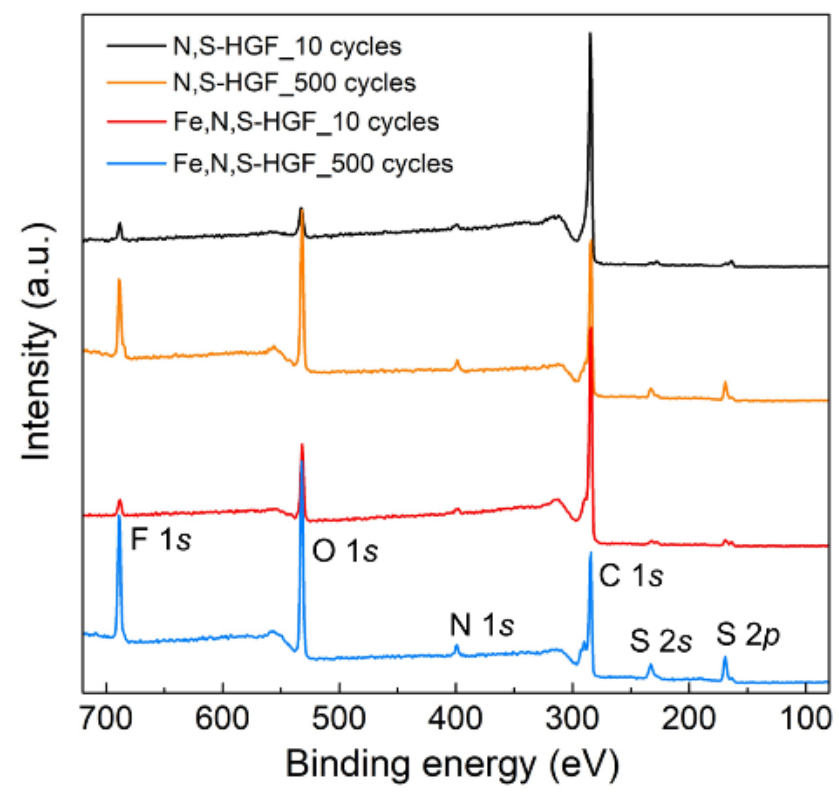

Supplementary Figure 13 | XPS survey spectra of post-cycling N,S-HGF and Fe,N,S-HGF electrodes. Although the pretreatment in the DME solvent for 24 h is applied for post-cycling electrodes, the distinct peak of F signal is detected and the peak intensities of O, N, S, F signals increase with the cycling, which suggests the attachment of LiTFSI or SEI formation in post-cycling electrodes. (a.u. represents arbitrary units)

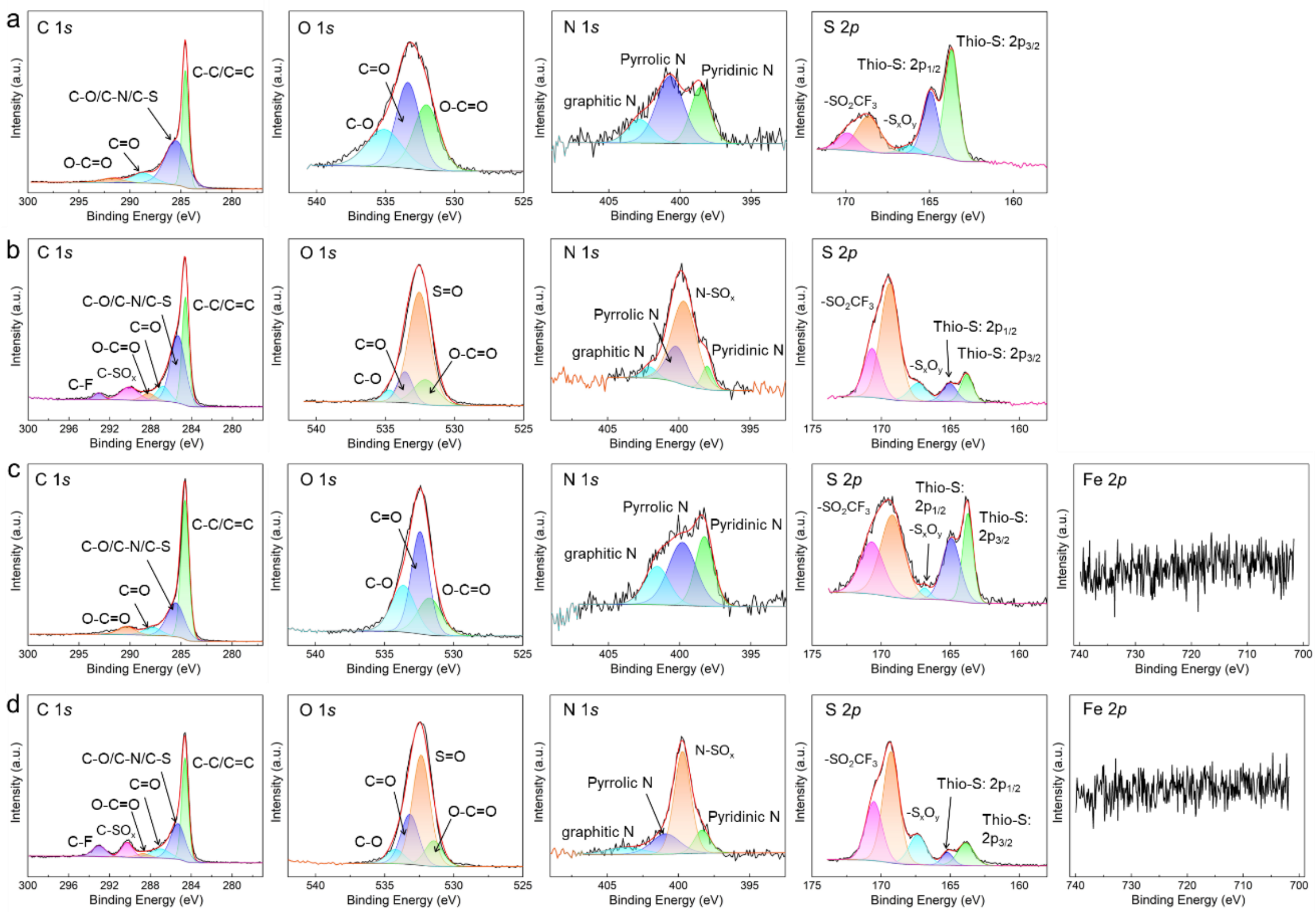

e

at%	C	O	N	S	Fe
N,S-HGF 10 cycles	92.80	4.73	1.43	1.04	/
N,S-HGF 500 cycles	88.00	9.88	1.10	1.02	/
Fe,N,S-HGF 10 cycles	88.42	9.39	1.39	0.80	/
Fe,N,S-HGF 500 cycles	85.55	12.10	1.28	1.07	/

Supplementary Figure 14 | XPS analysis of post-cycling N,S-HGF and Fe,N,S-HGF electrodes. **a**,**b**, High-resolution XPS spectra of the N,S-HGF electrode cycled for 10 cycles (**a**) and 500 cycles (**b**), respectively. **c**,**d**, High-resolution XPS spectra of the Fe,N,S-HGF electrode cycled for 10 cycles (**c**) and 500 cycles (**d**), respectively. C 1*s*, O 1*s*, N 1*s*, S 2*p*, or Fe 2*p* spectra are presented, including peak deconvolution and assignments. (a.u. represents arbitrary units) **e**, A summary of atomic ratios of C, O, N, S, or Fe elements for post-cycling N,S-HGF and Fe,N,S-HGF electrodes based on the peak deconvolution and assignments. Note that the peaks assigned to LiTFSI are excluded for the ratio calculation. For the post-cycling N,S-HGF and Fe,N,S-HGF electrodes, XPS survey spectra show the distinct signal for F element and enhanced O, N, and S signals with the cycling, suggesting the attachment of LiTFSI in the HGF electrode. Through the peak deconvolution and assignment in the high-resolution XPS spectra of N,S-HGF and Fe,N,S-HGF electrodes after 10 and 500 cycles, we found that the content and bond environment of N and S dopants remained similar as that of pristine electrodes, demonstrating the electrochemical stability of heteroatom dopants in N,S-HGF and Fe,N,S-HGF electrodes during the electrochemical cycling.

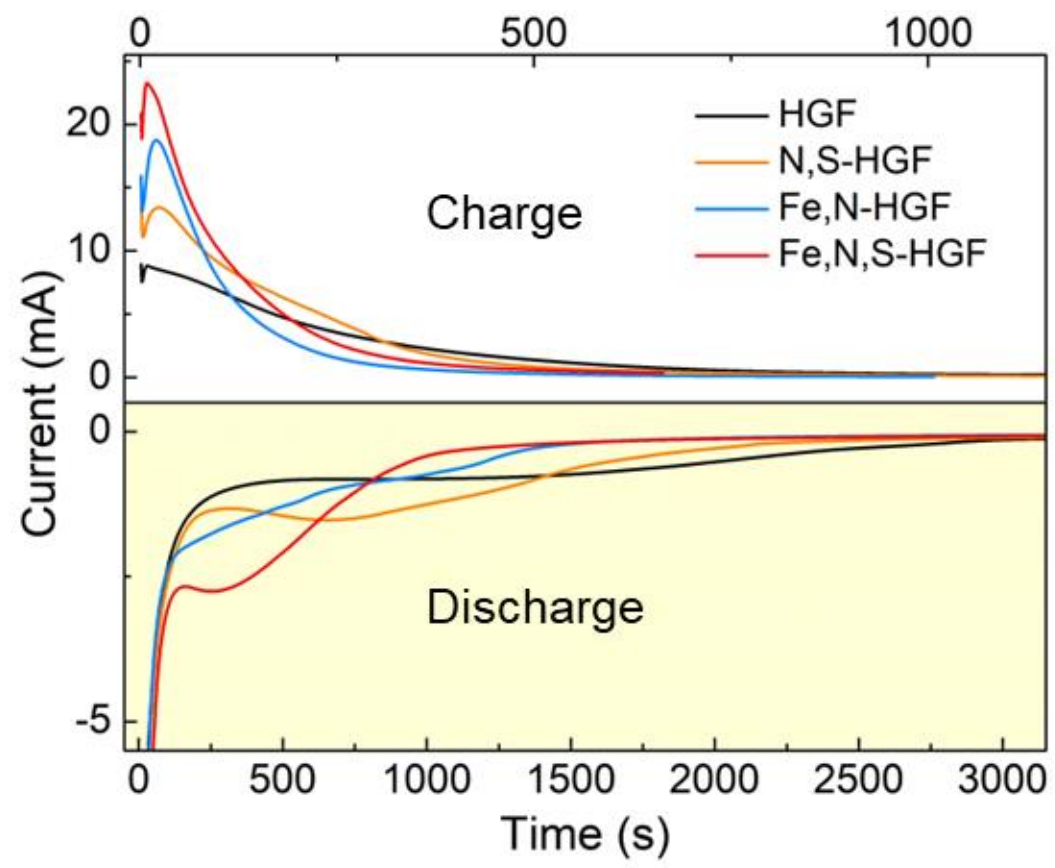

Supplementary Figure 15 | Representative chronoamperometry profiles. Chronoamperometry curves (current vs. time) of heteroatom-doped HGFs when applying the voltage of 2.8 or 1.8 V, respectively.

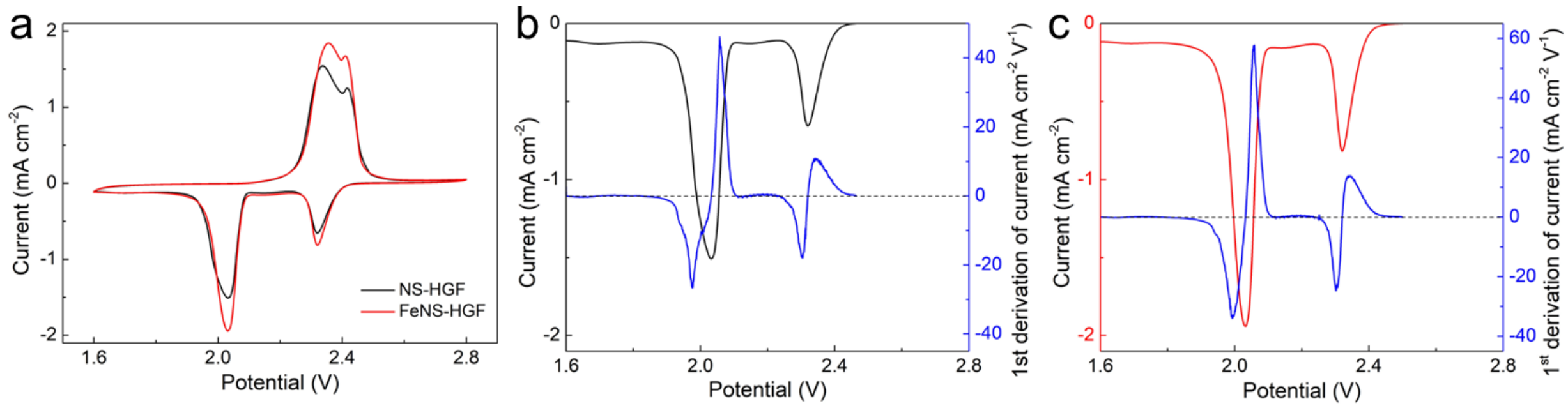

Supplementary Figure 16 | Charge number calculation based on cyclic voltammetry (CV) for N,S-HGF and Fe,N,S-HGF. a, CV curves of the N,S-HGF and Fe,N,S-HGF at the scan rate of 0.05 mV s^{-1}. **b**,**c**, CV and first derivative of current density versus voltage of the N,S-HGF (**b**) and Fe,N,S-HGF (**c**). The electron number transferred for the electrochemical steps in sulfur reduction reaction (SRR) can be calculated from CV curves. In the integration of CV curves for electron transfer number, all the negative current is calculated, including both the current below zero during discharge and the negative current in the initial period of charge. The charge number calculated from the integrated area of CV curves is compared with total charge number and converted to the formally electron transfer number in a 16-electron process. Voltage ranges for the integration of CV curves are determined by the first derivative of current density. Electron transfer numbers for each voltage region (1st peak, middle, 2nd peak) are analyzed to deduce the reaction intermediates.

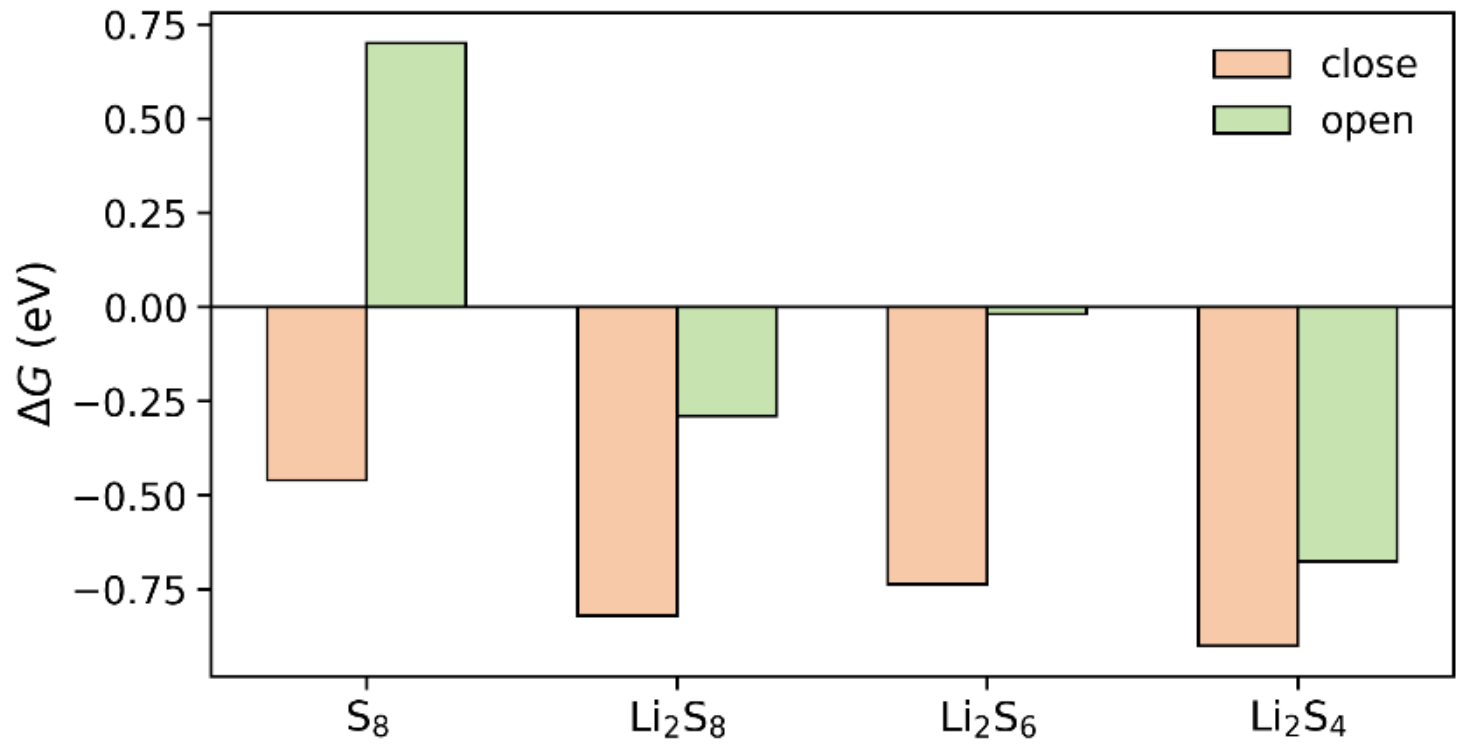

Supplementary Figure 17 | Comparison of close-shell and open-shell polysulfide adsorption on Fe sites. The close-shell polysulfides show the stronger adsorption compared to open-shell structures.

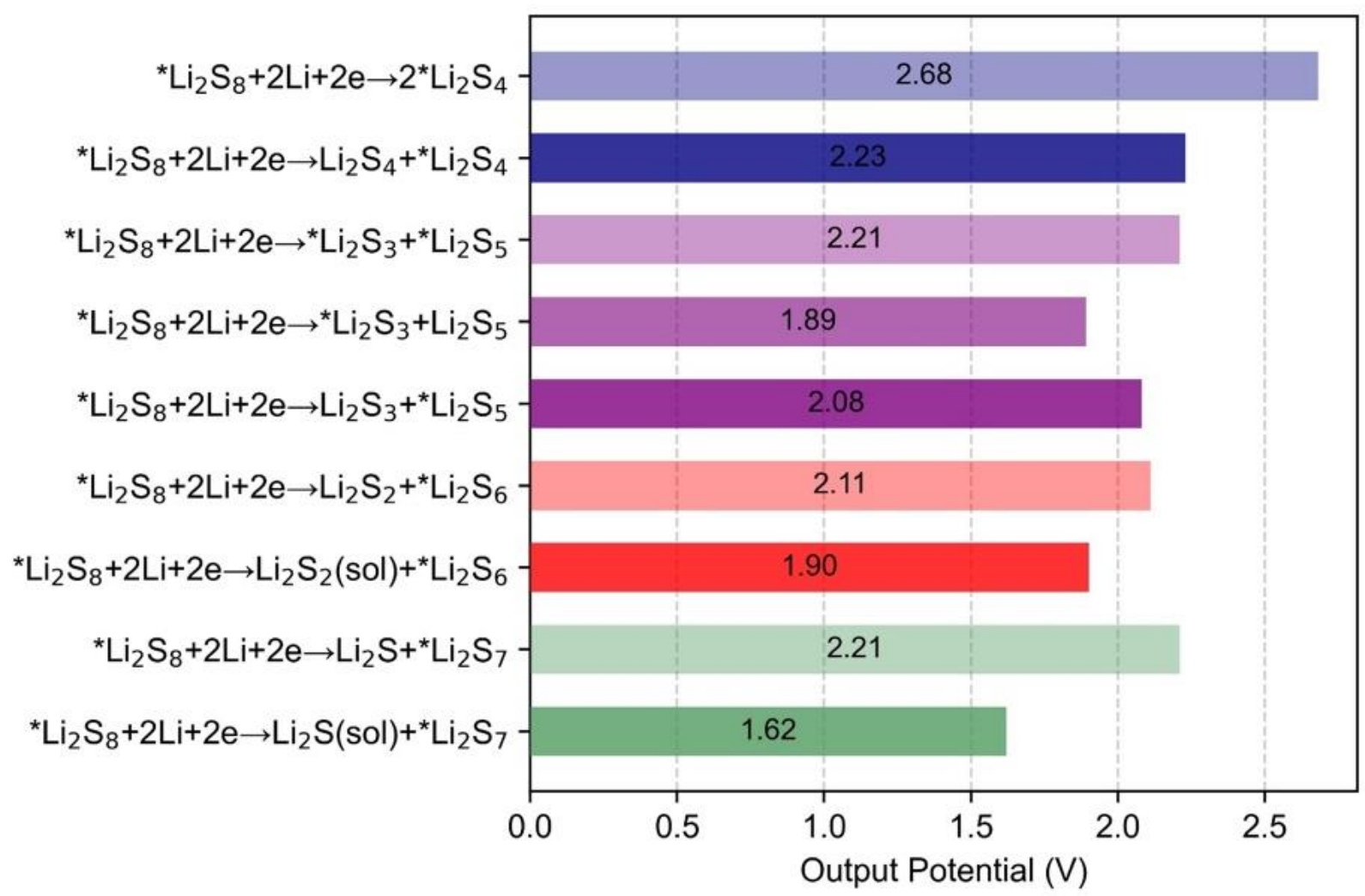

Supplementary Figure 18 | Calculated output potential for $*Li_2S_8$ reduction. The adsorbed $*Li_2S_8$ reduction to Li_2S_7, Li_2S_6, Li_2S_4, Li_2S_3 in molecule state or in adsorbed state. Considering the fast kinetic of $*Li_2S_8$ formation from S_8, we assume that the Fe site is limited and being primarily occupied by $*Li_2S_8$. Only forming one adsorbed species is considered in the main text.

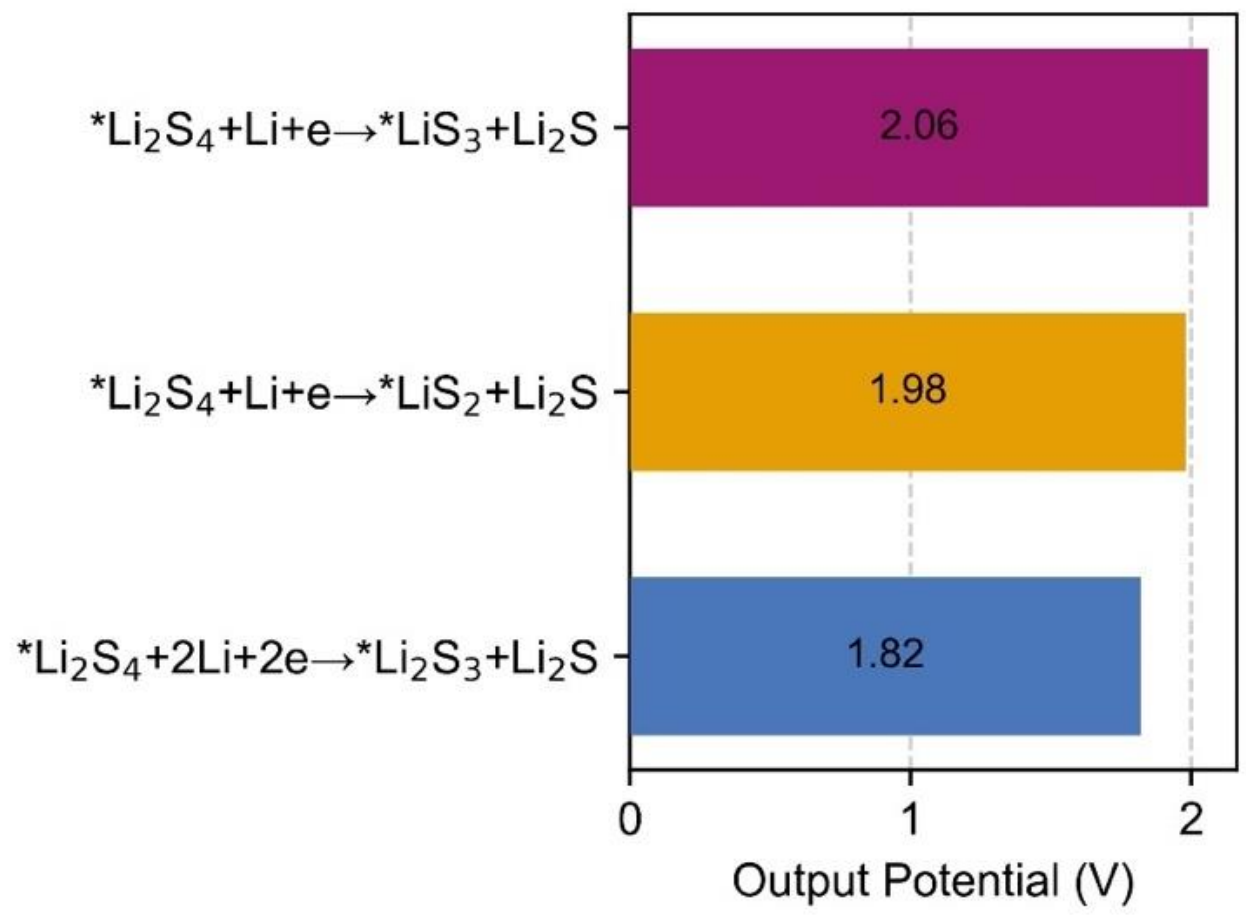

Supplementary Figure 19 | Calculated output potential for *Li_2S_4 reduction on Fe site. The adsorbed Li_2S_4 will either undergo one-electron transfer to *LiS_3 or LiS_2, or undergo two-electron transfer to *Li_2S_3.

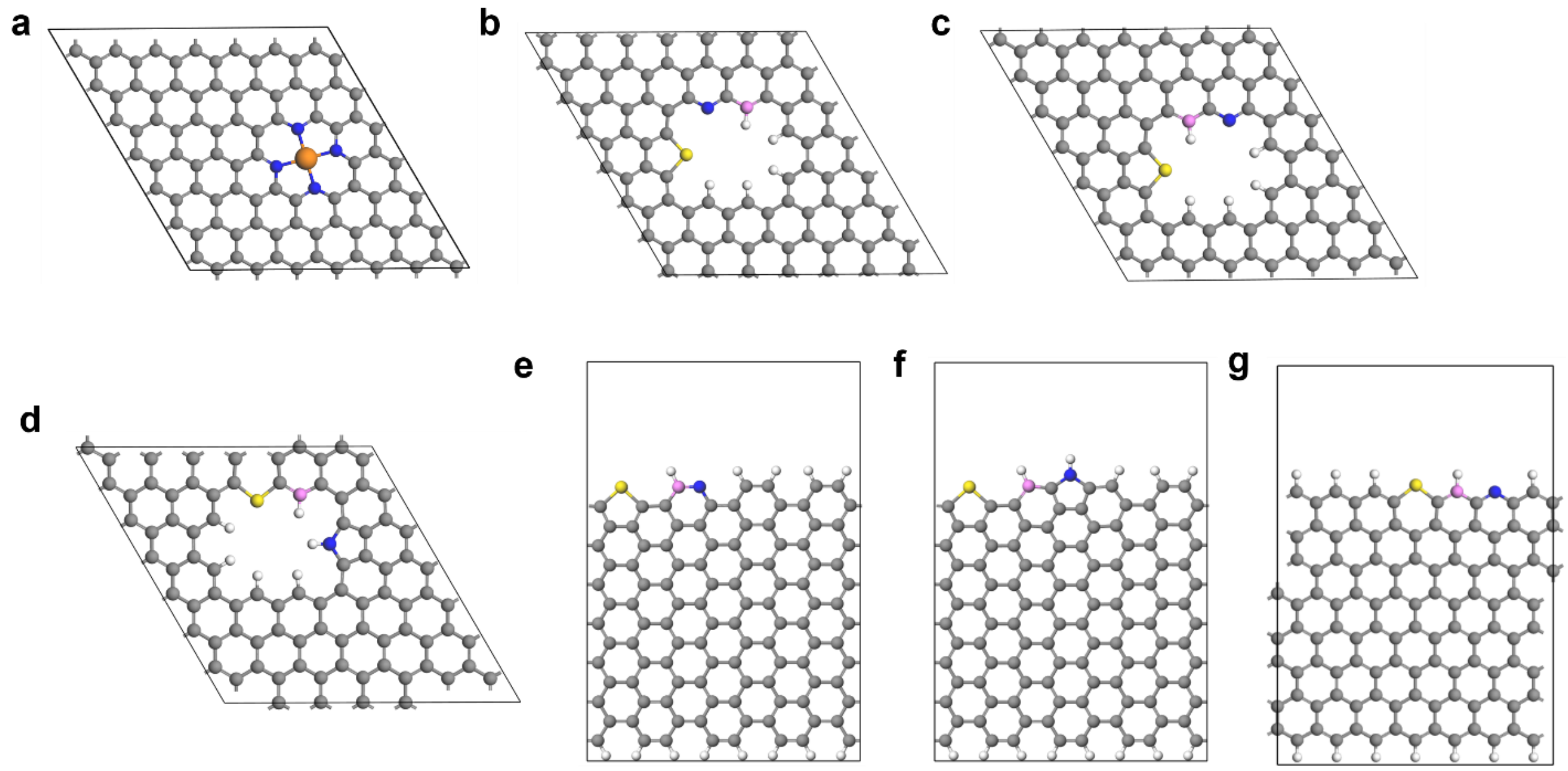

Supplementary Figure 20 | Atomic structures of N,S-codoped graphene models and Fe-N_x model used in this study. Upper column: **a**, Fe-N_4, **b**, defect-1, **c**, defect-2. Lower column: **d**, defect-3, **e**, armchair-1, **f**, armchair-2, **g**, zigzag-1. Color scheme: grey: C, white: H, pink: C chosen to be the interacting site, blue: N, yellow: S.

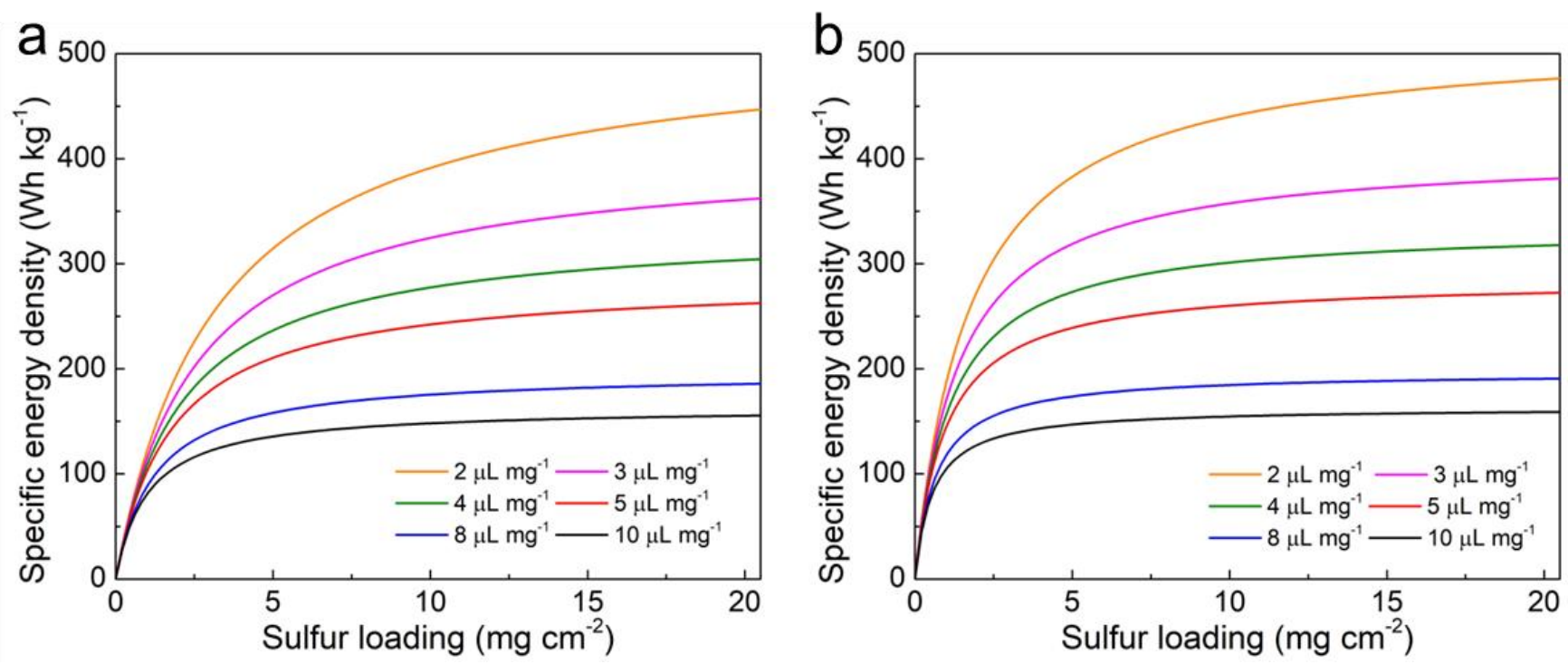

Supplementary Figure 21 | Analysis of the effect of sulfur loading and E/S ratio on the energy density of Li-S batteries. a, Analytic model of single-sided electrode configuration showing specific energy density versus sulfur loading at various E/S ratios. **b**, Analytic model of double-sided electrode configuration showing specific energy density versus sulfur loading at various E/S ratios. The analytical model includes the total weight (without Al-plastic pack) and is based on the following assumptions: (i) The positive electrode is considered to be a blade-cast sulfur-carbon composite with a sulfur mass ratio of 70wt% on an Al current collector (10 µm, 8.96 g cm^{-3}); Li-metal negative electrode is considered to be dense Li coated onto a Cu current collector (10 µm, 2.70 g cm^{-3}), and the N/P ratio is 1; Celgard2500 is used as the separator (25 µm, 0.57 g cm^{-3}), (ii) The specific capacity and nominal discharge voltage are 1000 mAh g^{-1} and 2.1 V, respectively; The density of electrolyte is considered to be 1.1 g cm^{-3}.

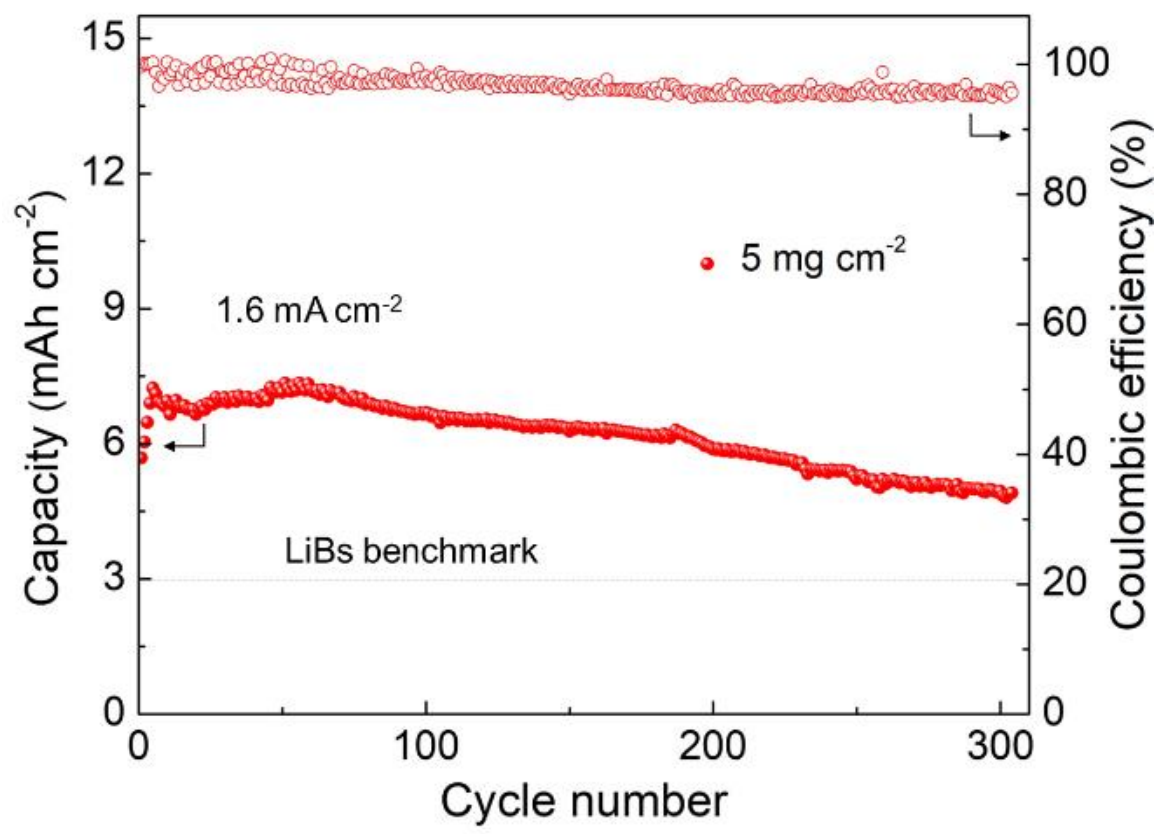

Supplementary Figure 22 | Cycling stability of the Fe,N,S-HGF-based sulfur electrodes. It was cycled at the current density of 1.6 mA cm^{-2} with the sulfur loading of 5 mg cm^{-2} at an E/S ratio of ~5 µL mg^{-1}.

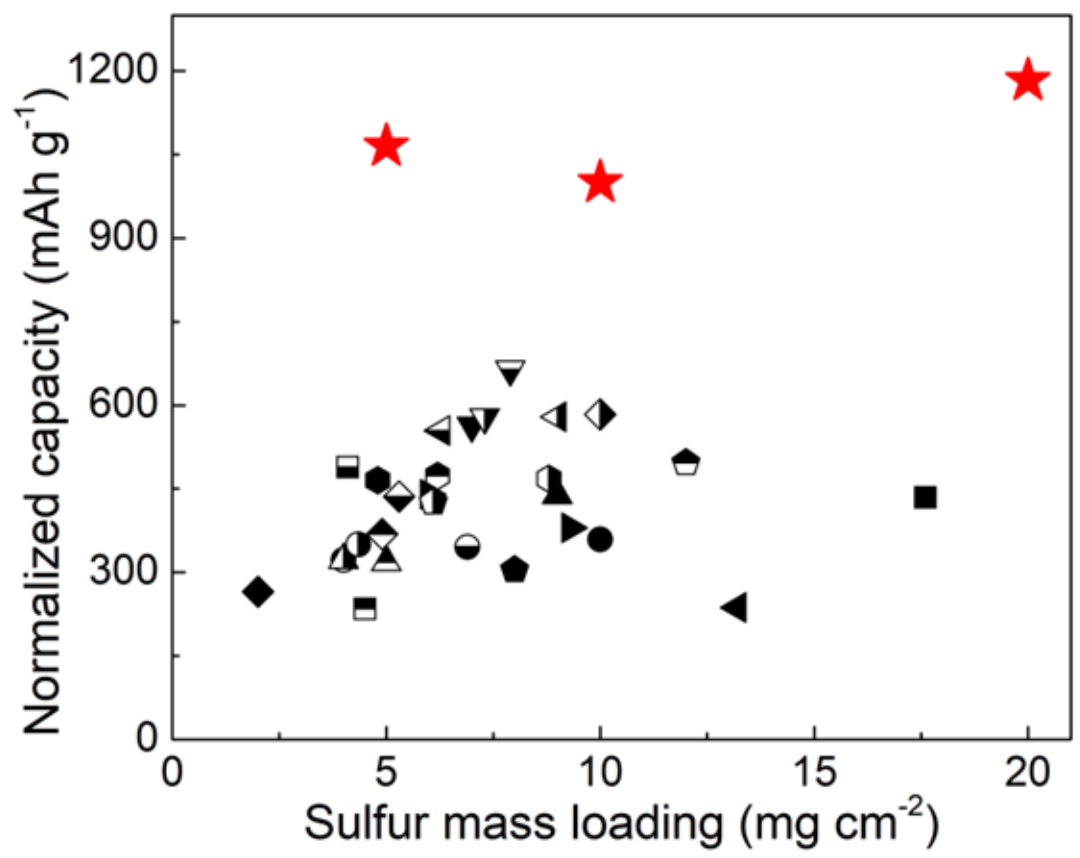

Supplementary Figure 23 | Comparison of normalized gravimetric capacity versus sulfur mass loading between previously reported sulfur electrodes and this work. The details can be found in Supplementary Table 4. Gravimetric capacity is normalized by the total mass of sulfur electrode including sulfur active material, catalyst, carbon additive, binder, and current collector.

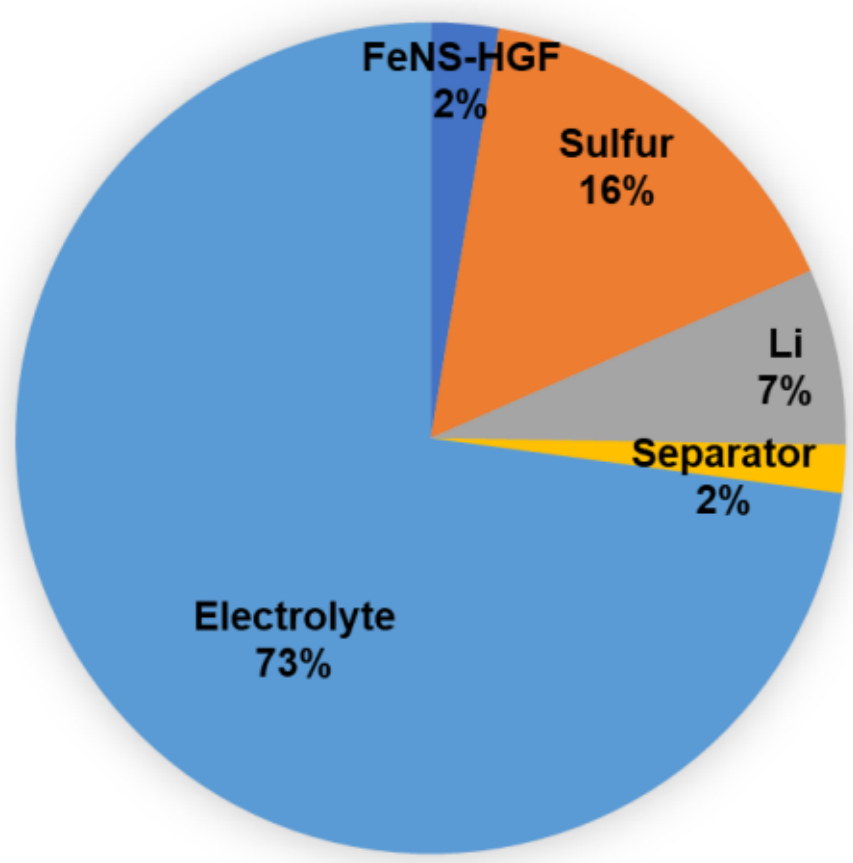

Component	Mass (mg)
FeNS-HGF	10
Sulfur	60
Li metal anode	26.04
Separator	7.125
Electrolyte	277.2
Total	380.365

Supplementary Figure 24 | The mass ratio pie chart of the lab-assembled Li-S pouch cell. As the Fe,N,S-HGF based electrode is free-standing, the Al & Cu foils are not included in the total weight. The N/P ratio is assumed to be 1.

Supplementary Table 1 | Comparisons of transport properties in different electrode materials and electrolytes.

Materials or Electrolyte	Li^+ diffusion coefficient ($cm^2\ s^{-1}$)	Electrical conductivity ($S\ cm^{-1}$)	Ion conductivity ($S\ cm^{-1}$)
$LiCoO_2$	10^{-10} to 10^{-8}	10^{-4}	10^{-8} to 10^{-6}
$LiMn_2O_4$	10^{-11} to 10^{-9}	10^{-6}	10^{-7} to 10^{-6}
$LiFePO_4$	10^{-15} to 10^{-14}	10^{-9}	10^{-16}
Graphite	10^{-14} to 10^{-8}	10^{2} to 10^{4}	
Sulfur	10^{-12}	5×10^{-30}, 5×10^{-18}	10^{-9}
Li_2S	10^{-15}	10^{-13}	7.51×10^{-11}, 10^{-13}
$LiPF_6$ EC/DMC	10^{-7} to 10^{-6}		10.7×10^{-3}
LiTFSI DME/DOL	4.3×10^{-6}		11×10^{-3}

The diffusion coefficient and conductivity numbers listed are obtained from these references[1-9].

Supplementary Table 2 | Charge number calculation results based on cyclic voltammetry for N,S-HGF and Fe,N,S-HGF.

Voltage range (V) vs. Li/Li$^+$	2nd peak	Middle	1st peak	Total
Electron transfer number (NS-HGF#1)	10.49	0.89	2.64	14.02
Formally electron transfer number (N,S-HGF#1)	11.98	1.01	3.01	16.00
Electron transfer number (NS-HGF#2)	10.89	1.01	2.83	14.73
Formally electron transfer number (N,S-HGF#2)	11.83	1.09	3.08	16.00
Electron transfer number (NS-HGF#3)	9.19	0.80	2.24	12.23
Formally electron transfer number (N,S-HGF#3)	12.02	1.05	2.93	16.00
Electron transfer number (FeNS-HGF#1)	11.60	0.85	3.20	15.65
Formally electron transfer number (Fe,N,S-HGF#1)	11.86	0.87	3.27	16.00
Electron transfer number (FeNS-HGF#2)	11.60	0.83	3.06	15.49
Formally electron transfer number (Fe,N,S-HGF#2)	11.98	0.86	3.16	16.00
Electron transfer number (FeNS-HGF#3)	12.77	0.82	3.37	16.96
Formally electron transfer number (Fe,N,S-HGF#3)	12.05	0.77	3.18	16.00

Supplementary Table 3 | Comparison of calculated output potentials for the first step of *Li_2S_4 reduction

Reactions	Output Potential (V)						
	Fe	Defect-1	Defect-2	Defect-3	Armchair-1	Armchair-2	zigzag
*Li_2S_4+Li+e → *$LiS_{2\ (Fe)}$+ Li_2S_2	1.98						
*Li_2S_4+Li+e → *$LiS_{2\ (C)}$+ Li_2S_2		1.49	1.67	0.53	1.76	2.07	0.81
*Li_2S_4+Li+e + Li_2S_4 → *$LiS_{2\ (C)}$ + Li_2S_2 + *Li_2S_4		2.39	2.57	1.44	2.66	2.97	1.71
*Li_2S_4+Li+e + Li_2S_8 → *$LiS_{2\ (C)}$ + Li_2S_2 +*Li_2s_8		2.31	2.49	1.36	2.58	2.89	1.63
*Li_2S_4+Li+e → *$LiS_{3\ (Fe)}$+ Li_2S	2.06						
*Li_2S_4+Li+e → *$LiS_{3\ (C)}$+ Li_2S		1.73	1.42	0.84	1.53	2.29	1.02
*Li_2S_4+Li+e + Li_2S_4 → *$LiS_{3\ (C)}$ + Li_2S + *Li_2S_4		2.63	2.32	1.74	2.43	3.20	1.92
*Li_2S_4+Li+e + Li_2S_8 → *$LiS_{3\ (C)}$ + Li_2S +*Li_2s_8		2.55	2.24	1.66	2.35	3.12	1.84

Supplementary Table 4 | Comparison of performance parameters of reported catalysts in high-loading and lean-electrolyte Li-S batteries.

Catalyst or host materials	Sulfur content (%)	S wt.% of electrode (%)	Gravimetric capacity[a] (mAh g^{-1})	S loading (mg cm^{-2})	E/S ratio (μL mg^{-1})	Areal capacity (mAh cm^{-2})	Cycle number	Capacity retention (%)	Ref.
Bi_2S_3	82.8	62	434.3	17.6	7.5	13.5	65	67	1[10]
CoZn/carbon	80	72	358.9	10	4.8	8	200	42	2[11]
3D-omsh/ZnS,Co-N-C	78.2	62.5	438.6	9	10	7.5	100	86.7	3[12]
$Co_{0.125}Zn_{0.875}S$	90	72	563.5	7	5	7	200	47	4[13]
CNT/$NiSe_2$	70	49	265.4	2	N/A	1.8	300	66.7	5[14]
$3VO_2$-1VN/G	77.2	61.8	236.9	13.2	7.5	5.7	20	67	6[15]
Lightweight MgB_2	75	60	379.8	9.3	6.5	7.9	100	75	7[16]
Ni_3FeN/G	70	63	465.2	4.8	4.7	4.8	25	62	8[17]
PCNF/S/BPQD	75	60	304.4	8	6.5	4.88	140	77	9[18]
Fe_2O_3-Fe_3C	74.8	59.8	234.7	4.5	10	2.4	100	90	10[19]
TiO@C-HS	70	49	322.2	4	40	3.5	150	67.7	11[20]
SAV@NG	80	68	318.3	5	N/A	3.2	200	75.2	12[21]
Ni@NG	N/A	N/A	--	6	10.5	3.02	100	80	13[22]
CoSA-N-C	74.2	55.7	368.8	4.9	10.5	4.24	120	81	14[23]
NC@SA-Co	70	56	--	7.2	N/A	8.3	100	51	15[24]
Co-N/G	90	67.5	440.1	6	12	5.1	100	90	16[25]
HMSC	N/A	45	473.4	6.2	2.4	7.8	100	64	17[26]
CNF-GA	70	63	496.6	12	N/A	10.8	30	65	18[27]
C@Fe_3O_4	80	64	--	5.5	N/A	6.07	200	77	19[28]
$CoSe_2$/G	70	39.7	350.0	4.35	15	4.78	100	75.7	20[29]
$NiCo_2O_4$ nanofiber	70	49	322.2	4	N/A	3.5	100	50	21[30]
TiO_2-MXene	75	60	578.5	7.3	15	8.6	200	42	22[31]
VS_4@RGO	70	70	583.8	10	10	8.34	100	83	23[32]
CC@CS@HPP	70	56	--	5.6	10	5	30	82	24[33]
In_2O_3-G-CNT	70	66.5	579.0	9	8	9.4	100	56	25[34]
Fe-Ni alloy	86	73	--	6.4	8	6.1	100	82	26[35]
$NiSe_2$	70	63	468.0	8.8	6	7.8	100	70	27[36]
MoB	90	72	426.1	6.1	7	4.76	200	75	28[37]
Co_4N/NG	70	63	488.7	4.1	12	4.5	100	70	29[38]
$Ni_3ZnC_{0.7}$/Ni	80	70	345.6	6.9	5.8	4.34	80	70	30[39]
SA-Cu@NCNF	90	66.5	--	10	10	8.44	100	76	31[40]
ZnS-SnS@NC	80	64	664.7	7.9	6	10	200	82	32[41]
SA-Zn-MXene	N/A	56	435.7	5.3	N/A	5.3	40	83	33[42]
S@N-$MoSe_{2-x}$	80	60	554.4	6.28	6.37	7.3	35	83	34[43]
V_8C_7-VO_2	77	61.5	--	9.2	5.2	7.3	100	62	35[44]
Co4W18/rGO	70	49	--	5.6	4.5	4.55	50	85	36[45]
Fe,N,S-HGF	75	75	1065	5	5	7.1	100/200/300	94/85/72	This work
Fe,N,S-HGF	83.3	83.3	999.6	10	5	12	100/200/300	100/95/86	This work
Fe,N,S-HGF	91	91	1183	20	5	26	80	86	This work

[a] Gravimetric capacity is based on the total mass of sulfur electrode including sulfur active material, catalyst, carbon additive, binder, and current collector.

Supplementary References

1 Park, M., Zhang, X., Chung, M., Less, G. B. & Sastry, A. M. A review of conduction phenomena in Li-ion batteries. *J. Power Sources* **195**, 7904-7929 (2010).

2 Valøen, L. O. & Reimers, J. N. Transport Properties of LiPF6-Based Li-Ion Battery Electrolytes. *J. Electrochem. Soc.* **152**, A882 (2005).

3 Zhang, K., Wang, L., Hu, Z., Cheng, F. & Chen, J. Ultrasmall Li2S Nanoparticles Anchored in Graphene Nanosheets for High-Energy Lithium-Ion Batteries. *Scientific Reports* **4**, 6467 (2014).

4 Liu, M. *et al.* Quantification of the Li-ion diffusion over an interface coating in all-solid-state batteries via NMR measurements. *Nat. Commun.* **12**, 5943 (2021).

5 Choi, S., Yoon, I., Nichols, W. T. & Shin, D. Carbon-coated Li2S cathode for improving the electrochemical properties of an all-solid-state lithium-sulfur battery using Li2S-P2S5 solid electrolyte. *Ceram. Int.* **44**, 7450-7453 (2018).

6 Tanibata, N. *et al.* High Formability and Fast Lithium Diffusivity in Metastable Spinel Chloride for Rechargeable All-Solid-State Lithium-Ion Batteries. *Advanced Energy and Sustainability Research* **1**, 2000025 (2020).

7 Lama, F. L., Marangon, V., Caballero, Á., Morales, J. & Hassoun, J. Diffusional Features of a Lithium-Sulfur Battery Exploiting Highly Microporous Activated Carbon. *ChemSusChem* **16**, e202202095 (2023).

8 Shan, Y. & Li, L. Limiting sulfur and mastering the diffusion of lithium ions with cerium oxide-based porous carbon rods. *Chem. Commun.* **57**, 9550-9553 (2021).

9 Zhang, F., Luo, Y., Gao, X. & Wang, R. Copolymerized Sulfur with Intrinsically Ionic Conductivity, Superior Dispersibility, and Compatibility for All-Solid-State Lithium Batteries. *ACS Sustainable Chemistry & Engineering* **8**, 12100-12109 (2020).

10 Hua, W. *et al.* Optimizing the p charge of S in p-block metal sulfides for sulfur reduction electrocatalysis. *Nature Catalysis* **6**, 174-184 (2023).

11 Li, H. *et al.* Developing high-power Li||S batteries via transition metal/carbon nanocomposite electrocatalyst engineering. *Nat. Nanotechnol.* **19**, 792-799 (2024).

12 Zhao, C. *et al.* A high-energy and long-cycling lithium–sulfur pouch cell via a macroporous catalytic cathode with double-end binding sites. *Nat. Nanotechnol.* **16**, 166-173 (2021).

13 Shen, Z. *et al.* Cation-doped ZnS catalysts for polysulfide conversion in lithium–sulfur batteries. *Nature Catalysis* **5**, 555-563 (2022).

14 Han, Z. *et al.* Machine-learning-assisted design of a binary descriptor to decipher electronic and structural effects on sulfur reduction kinetics. *Nature Catalysis* **6**, 1073-1086 (2023).

15 Song, Y. *et al.* Synchronous immobilization and conversion of polysulfides on a VO2–VN binary host targeting high sulfur load Li–S batteries. *Energy & Environmental Science* **11**, 2620-2630 (2018).

16 Pang, Q., Kwok, C. Y., Kundu, D., Liang, X. & Nazar, L. F. Lightweight Metallic MgB2 Mediates Polysulfide Redox and Promises High-Energy-Density Lithium-Sulfur Batteries. *Joule* **3**, 136-148 (2019).

17 Zhao, M. *et al.* Activating Inert Metallic Compounds for High-Rate Lithium–Sulfur Batteries Through In Situ Etching of Extrinsic Metal. *Angewandte Chemie International Edition* **58**, 3779-3783 (2019).

18 Xu, Z.-L. *et al.* Exceptional catalytic effects of black phosphorus quantum dots in shuttling-free lithium sulfur batteries. *Nat. Commun.* **9**, 4164 (2018).

19 Cao, Z. *et al.* Integrating Polar and Conductive Fe2O3–Fe3C Interface with Rapid Polysulfide Diffusion and Conversion for High-Performance Lithium–Sulfur Batteries. *ACS Appl. Mater. Interfaces* **11**, 39772-39781 (2019).

20 Li, Z. *et al.* A sulfur host based on titanium monoxide@carbon hollow spheres for advanced lithium–sulfur batteries. *Nat. Commun.* **7**, 13065 (2016).

21 Zhou, G. *et al.* Theoretical Calculation Guided Design of Single-Atom Catalysts toward Fast Kinetic and Long-Life Li–S Batteries. *Nano Lett.* **20**, 1252-1261 (2020).

22 Zhang, L. *et al.* Single Nickel Atoms on Nitrogen-Doped Graphene Enabling Enhanced Kinetics of Lithium–Sulfur Batteries. *Adv. Mater.* **31**, 1903955 (2019).

23 Li, Y. *et al.* Fast conversion and controlled deposition of lithium (poly)sulfides in lithium-sulfur batteries using high-loading cobalt single atoms. *Energy Storage Materials* **30**, 250-259 (2020).

24 Li, Y. *et al.* A Freestanding Flexible Single-Atom Cobalt-Based Multifunctional Interlayer toward Reversible and Durable Lithium-Sulfur Batteries. *Small Methods* **4**, 1900701 (2020).

25 Du, Z. *et al.* Cobalt in Nitrogen-Doped Graphene as Single-Atom Catalyst for High-Sulfur Content Lithium–Sulfur Batteries. *J. Am. Chem. Soc.* **141**, 3977-3985 (2019).

26 Xue, W. *et al.* Intercalation-conversion hybrid cathodes enabling Li–S full-cell architectures with jointly superior gravimetric and volumetric energy densities. *Nature Energy* **4**, 374-382 (2019).

27 Tu, S. *et al.* A Polysulfide-Immobilizing Polymer Retards the Shuttling of Polysulfide Intermediates in Lithium–Sulfur Batteries. *Adv. Mater.* **30**, 1804581 (2018).

28 He, J., Luo, L., Chen, Y. & Manthiram, A. Yolk–Shelled C@Fe3O4 Nanoboxes as Efficient Sulfur Hosts for High-Performance Lithium–Sulfur Batteries. *Adv. Mater.* **29**, 1702707 (2017).

29 Yuan, H. *et al.* Conductive and Catalytic Triple-Phase Interfaces Enabling Uniform Nucleation in High-Rate Lithium–Sulfur Batteries. *Advanced Energy Materials* **9**, 1802768 (2019).

30 Liu, Y.-T. *et al.* NiCo2O4 Nanofibers as Carbon-Free Sulfur Immobilizer to Fabricate Sulfur-Based Composite with High Volumetric Capacity for Lithium–Sulfur Battery. *Advanced Energy Materials* **9**, 1803477 (2019).

31 Jiao, L. *et al.* Capture and Catalytic Conversion of Polysulfides by In Situ Built TiO2-MXene Heterostructures for Lithium–Sulfur Batteries. *Advanced Energy Materials* **9**, 1900219 (2019).

32 Luo, L., Li, J., Yaghoobnejad Asl, H. & Manthiram, A. In-Situ Assembled VS4 as a Polysulfide Mediator for High-Loading Lithium–Sulfur Batteries. *ACS Energy Letters* **5**, 1177-1185 (2020).

33 Ye, Z., Jiang, Y., Li, L., Wu, F. & Chen, R. A High-Efficiency CoSe Electrocatalyst with Hierarchical Porous Polyhedron Nanoarchitecture for Accelerating Polysulfides Conversion in Li–S Batteries. *Adv. Mater.* **32**, 2002168 (2020).

34 Hua, W. *et al.* Selective Catalysis Remedies Polysulfide Shuttling in Lithium-Sulfur Batteries. *Adv. Mater.* **33**, 2101006 (2021).

35 He, J., Bhargav, A. & Manthiram, A. High-Energy-Density, Long-Life Lithium–Sulfur Batteries with Practically Necessary Parameters Enabled by Low-Cost Fe–Ni Nanoalloy Catalysts. *ACS Nano* **15**, 8583-8591 (2021).

36 Shao, A. H. *et al.* Ultrathin Nanosheet-Assembled Flowerlike NiSe2 Catalyst Boosts Sulfur Redox Reaction Kinetics for Li–S Batteries. *ACS Applied Energy Materials* **4**, 3431-3438 (2021).

37 He, J., Bhargav, A. & Manthiram, A. Molybdenum Boride as an Efficient Catalyst for Polysulfide Redox to Enable High-Energy-Density Lithium–Sulfur Batteries. *Adv. Mater.* **32**, 2004741 (2020).

38 Zhao, M. *et al.* Electrochemical Phase Evolution of Metal-Based Pre-Catalysts for High-Rate Polysulfide Conversion. *Angewandte Chemie International Edition* **59**, 9011-9017 (2020).

39 Hong, X.-J. *et al.* Sulfophilic and lithophilic sites in bimetal nickel-zinc carbide with fast conversion of polysulfides for high-rate Li-S battery. *Chem. Eng. J.* **404**, 126566 (2021).

40 Xiao, R. *et al.* Electronic structure adjustment of lithium sulfide by a single-atom copper catalyst toward high-rate lithium-sulfur batteries. *Energy Storage Materials* **51**, 890-899 (2022).

41 Yao, W. *et al.* ZnS-SnS@NC Heterostructure as Robust Lithiophilicity and Sulfiphilicity Mediator toward High-Rate and Long-Life Lithium–Sulfur Batteries. *ACS Nano* **15**, 7114-7130 (2021).

42 Zhang, D. *et al.* Catalytic Conversion of Polysulfides on Single Atom Zinc Implanted MXene toward High-Rate Lithium–Sulfur Batteries. *Adv. Funct. Mater.* **30**, 2002471 (2020).

43 Shi, Z. *et al.* Manipulating Electrocatalytic Li2S Redox via Selective Dual-Defect Engineering for Li–S Batteries. *Adv. Mater.* **33**, 2103050 (2021).

44 Cai, J. *et al.* 3D Printing of a V8C7–VO2 Bifunctional Scaffold as an Effective Polysulfide Immobilizer and Lithium Stabilizer for Li–S Batteries. *Adv. Mater.* **32**, 2005967 (2020).

45 Lei, J. *et al.* Single-dispersed polyoxometalate clusters embedded on multilayer graphene as a bifunctional electrocatalyst for efficient Li-S batteries. *Nat. Commun.* **13**, 202 (2022).
