# Supplementary material for: Designing and mapping cascade catalysis pathway for balanced polysulfide conversion in Li-S batteries
Source: Nat Commun. 2026 Jul 1;17:8191. doi: 10.1038/s41467-026-74160-3 (PMC13462536; doi:10.1038/s41467-026-74160-3)
Supplement: Supplementary file 2 [file 41467_2026_74160_MOESM2_ESM.pdf]

Description of Additional Supplementary Files

File Name: Supplementary Data

Description: It is a ZIP archive containing the optimized computational structure files reported in this study. The individual file names indicate the adsorbate. Since all files are provided in the same computational format, no additional file-specific description is required beyond the file names.
