# Supplementary material for: Designing and mapping cascade catalysis pathway for balanced polysulfide conversion in Li-S batteries
Source: Nat Commun. 2026 Jul 1;17:8191. doi: 10.1038/s41467-026-74160-3 (PMC13462536; doi:10.1038/s41467-026-74160-3)
Supplement: Supplementary file 3 [file 41467_2026_74160_MOESM4_ESM.pdf]

nature portfolio

Peer Review File

Designing and mapping cascading catalysis pathway for balanced polysulfide conversion

Corresponding Author: Dr Xiangfeng Duan

This manuscript has been previously reviewed at another journal. This document only contains information relating to versions considered at Nature Communications.

This file contains all reviewer reports in order by version, followed by all author rebuttals in order by version.

Version 0:

Reviewer comments:

Reviewer #1

(Remarks to the Author)
I was reviewer #1. The authors have carefully revised the manuscript and provided responses to the reviewers’ comments. The experimental results are generally sufficient. Therefore, I recommend the manuscript for publication after the following issues are clarified.

1. Figure 1 mainly presents a conceptual schematic, while Figure 2 focuses on material characterization. As a result, the manuscript enters the key mechanistic discussion somewhat indirectly (or slow). It is suggested that Figure 1 be revised to more clearly illustrate the full cascading SRR reaction pathway, so that readers can more intuitively understand the reaction sequence and the respective roles of the Fe sites and N,S-C sites at the beginning of the manuscript.

2. The authors propose that reaction intermediates migrate from Fe sites to N,S-C sites, which is central to the proposed cascading catalysis mechanism. However, the experimental evidence supporting this intermediate migration is not sufficiently convincing. Additional clarification or stronger supporting evidence would help substantiate this claim.

Reviewer #2

(Remarks to the Author)
This manuscript presents a comprehensive and mechanistic study on cascading catalysis for the sulfur reduction reaction (SRR) in lithium–sulfur batteries. By combining advanced operando Raman spectroscopy, detailed electrochemical analysis, and DFT calculations, the authors successfully map out a full cascading reaction pathway and demonstrate the synergistic effect of Fe and N,S dual active sites. The work is well-structured, the data are of high quality, and the conclusions are supported by solid experimental and theoretical evidence. The authors have well addressed what I was puzzled. I recommend accepting publication after minor revisions.

1. The cycling performance of Fe,N,S-HGF presented in Fig.3f exhibited noticeable capacity fluctuations, which implied relatively unstable charging and discharging processes for Fe,N,S-HGF catalysts. The authors should provide a reasonable explanation for this phenomenon. Additionally, if possible, long-term cycling performance of other control samples could be provided to further highlight the enhanced cycling stability achieved by the target catalyst.
2. The figure legend should be consistent throughout all figures. For instance, the target catalyst Fe,N,S-HGF is labeled as "FeNS-HGF" in Fig.6a This discrepancy should be corrected. Please carefully check out…

Reviewer #3

(Remarks to the Author)
All my concerns have been addressed, and I recommend accepting this paper for Nature Communications.

Version 1:

Reviewer comments:

Reviewer #1

(Remarks to the Author)
The authors have carefully revised the manuscript, and I recommend it published on this journal with this version.

Reviewer #2

(Remarks to the Author)
No revision needed. accepted without revision.

Open Access This Peer Review File is licensed under a Creative Commons Attribution 4.0 International License, which permits use, sharing, adaptation, distribution and reproduction in any medium or format, as long as you give appropriate credit to the original author(s) and the source, provide a link to the Creative Commons license, and indicate if changes were made.
In cases where reviewers are anonymous, credit should be given to 'Anonymous Referee' and the source.
The images or other third party material in this Peer Review File are included in the article's Creative Commons license, unless indicated otherwise in a credit line to the material. If material is not included in the article's Creative Commons license and your intended use is not permitted by statutory regulation or exceeds the permitted use, you will need to obtain permission directly from the copyright holder.
To view a copy of this license, visit https://creativecommons.org/licenses/by/4.0/

REVIEWER COMMENTS

Reviewer #1:

I was reviewer #1. The authors have carefully revised the manuscript and provided responses to the reviewers' comments. The experimental results are generally sufficient. Therefore, I recommend the manuscript for publication after the following issues are clarified.

Response: Thanks for the reviewer's further comment and recommendation for publication. We have clarified the additional issues raised by the reviewer and made the revisions accordingly.

1. Figure 1 mainly presents a conceptual schematic, while Figure 2 focuses on material characterization. As a result, the manuscript enters the key mechanistic discussion somewhat indirectly (or slow). It is suggested that Figure 1 be revised to more clearly illustrate the full cascading SRR reaction pathway, so that readers can more intuitively understand the reaction sequence and the respective roles of the Fe sites and N,S-C sites at the beginning of the manuscript.

Response: Thanks for the excellent suggestion. To better present the cascading SRR pathway, we have modified Figure 1 to more clearly illustrate the respective role of Fe and N,S-C sites, as displayed below.

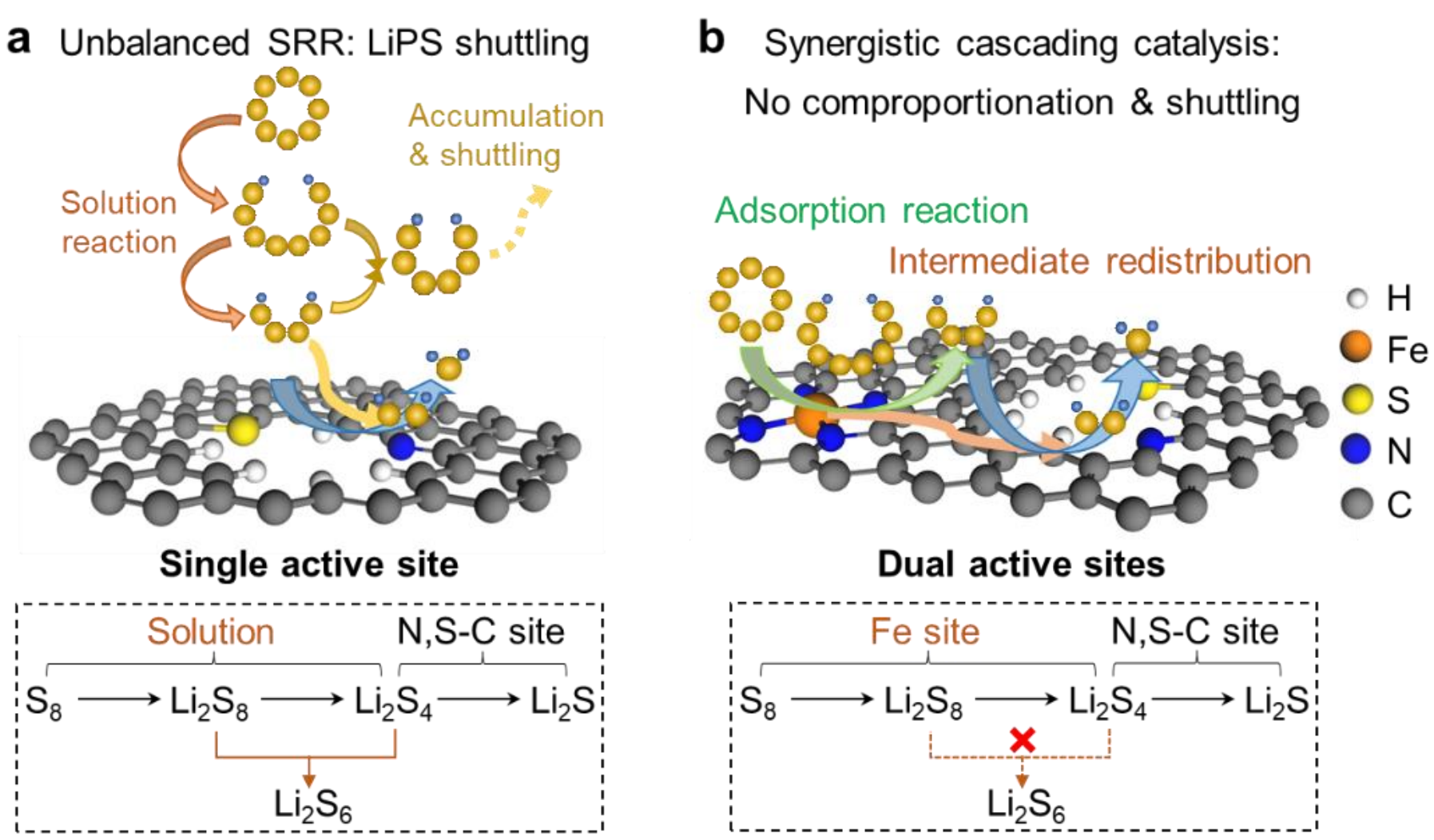

Figure R1 | Synergetic strategy of heteroatom-doped HGF host for SRR. **a**,**b**, Schematic illustration of single-site (**a**) and dual-site (**b**) electrocatalytic SRR process, highlighting the synergistic cascading catalysis on dual active sites. This cascading process retrains long-chain LiPSs on the catalyst surface, mitigating comproportionation reaction and the LiPS shuttling effect, while facilitating the on-surface migration of intermediates between active sites to efficiently transform LiPSs into the final product.

2. The authors propose that reaction intermediates migrate from Fe sites to N,S-C sites, which is central to the proposed cascading catalysis mechanism. However, the experimental evidence supporting this intermediate migration is not sufficiently convincing. Additional clarification or stronger supporting evidence would help substantiate this claim.

Response: We thank the reviewer for this valuable comment. We agree that direct experimental visualization of "intermediate migration" from Fe sites to N,S-C sites is challenging with currently available characterization techniques, and we appreciate the opportunity to clarify this point from a mechanistic perspective. In particular, we want to clarify that the concept of "migration of intermediates" in the manuscript should not be interpreted as a directly resolved physical diffusion pathway of a single adsorbed species from Fe sites to N,S-C sites. It is more appropriately

described as a thermodynamically driven site-transfer (or reaction-sequence) process enabled by the coexistence of Fe sites and N,S-C sites. Specifically, once Li_2S_4 is formed or captured at the Fe sites, the subsequent reduction does not proceed with all intermediates remaining on Fe sites. Rather, the most favorable pathway we identified is that Li_2S_4 at Fe sites undergoes further lithiation/electron-transfer steps to form $*LiS_3$ or $*LiS_2$ species that were stabilized on neighboring N,S-C sites, while Fe sites are released for further capturing higher-order polysulfides for sustained catalytic reduction. This thermodynamic preference indicates a cooperative division of roles between the two types of active sites: Fe sites are more effective for the initial capture/activation of higher-order polysulfides, whereas N,S-C sites act as active sites for stabilizing the more reduced intermediates derived from $*Li_2S_4$. Therefore, the term “migration” here should be understood as a redistribution of intermediates that reflects the cooperative involvement of two distinct active sites within an elementary step rather than the migration of a single intermediate. We acknowledge that the detailed kinetics and configurational evolution of such atomic level site-to-site transfer are beyond the ability of the present DFT calculations because of the large configurational space and length scales involved. Accordingly, we have modified relevant discussions in the manuscript to avoid potential misinterpretation.

Reviewer #2:

This manuscript presents a comprehensive and mechanistic study on cascading catalysis for the sulfur reduction reaction (SRR) in lithium–sulfur batteries. By combining advanced operando Raman spectroscopy, detailed electrochemical analysis, and DFT calculations, the authors successfully map out a full cascading reaction pathway and demonstrate the synergistic effect of Fe and N,S dual active sites. The work is well-structured, the data are of high quality, and the conclusions are supported by solid experimental and theoretical evidence. The authors have well addressed what I was puzzled. I recommend accepting publication after minor revisions.

Response: Thanks for the reviewer's further comment and recommendation for accepting publication. We have addressed the additional questions and revised accordingly.

1. The cycling performance of Fe,N,S-HGF presented in Fig.3f exhibited noticeable capacity fluctuations, which implied relatively unstable charging and discharging processes for Fe,N,S-HGF catalysts. The authors should provide a reasonable explanation for this phenomenon. Additionally, if possible, long-term cycling performance of other control samples could be provided to further highlight the enhanced cycling stability achieved by the target catalyst.

Response: We appreciate the reviewer's comment. The fluctuation in the cycling performance of Fe,N,S-HGF should be ascribed to the temperature fluctuation during the test period, as our battery testing system is not located in a room with independently controlled temperature. As suggested by the reviewer, we have also added the cycling performance of HGF and Fe,N-HGF samples in the supporting information, as shown below.

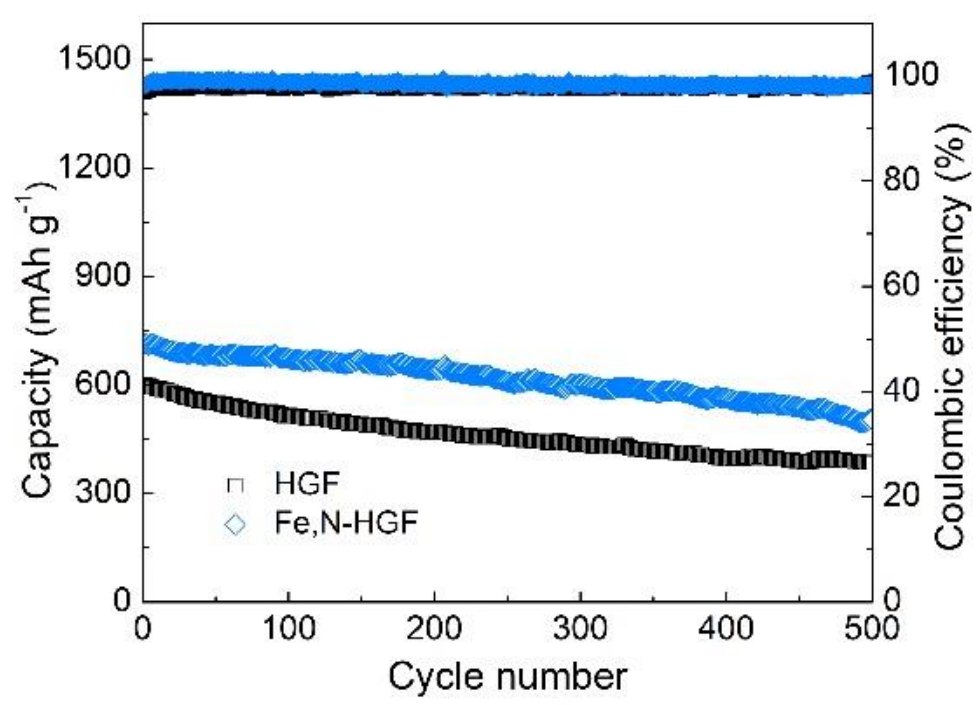

Figure R3 | Cycling performance of HGF and Fe,N-HGF based cathodes at a current density of 1 C.

2. The figure legend should be consistent throughout all figures. For instance, the target catalyst Fe,N,S-HGF is labeled as "FeNS-HGF" in Fig.6a This discrepancy should be corrected. Please carefully check out…

Response: Thanks for the careful review and suggestion. We have thoroughly gone through the manuscript and all figures to ensure the consistent description of sample names.

Reviewer #3:

All my concerns have been addressed, and I recommend accepting this paper for Nature Communications.

Response: We sincerely appreciate the reviewer's comment and recommendation for publication.
